# MAL2 reprograms lipid metabolism in intrahepatic cholangiocarcinoma via EGFR/SREBP-1 pathway based on single-cell RNA sequencing

**DOI:** 10.1038/s41419-024-06775-7

**Published:** 2024-06-12

**Authors:** Tian Huang, Hengsong Cao, Chuan Liu, Xiaohu Sun, Shipeng Dai, Li Liu, Yuliang Wang, Cheng Guo, Xuehao Wang, Yun Gao, Weiwei Tang, Yongxiang Xia

**Affiliations:** 1 Hepatobiliary Center, The First Affiliated Hospital of Nanjing Medical University; Key Laboratory of Liver Transplantation, Chinese Academy of Medical Sciences; NHC Key laboratory of Hepatobiliary cancers,Nanjing, China, Nanjing, Jiangsu China; 2 State Key Laboratory of Modern Chinese Medicine, Tianjin University of Traditional Chinese Medicine, Tianjin, China; 3 School of Basic Medicine, Nanjing Medical University, Nanjing, Jiangsu China; 4 Department of Otorhinolaryngology Head and Neck Surgery, Guangzhou First People’s Hospital, Guangzhou, Guangdong China

**Keywords:** Cancer metabolism, Cancer genomics

## Abstract

Intrahepatic cholangiocarcinoma (ICC) is a highly aggressive cancer characterized by a poor prognosis and resistance to chemotherapy. In this study, utilizing scRNA-seq, we discovered that the tetra-transmembrane protein mal, T cell differentiation protein 2 (MAL2), exhibited specific enrichment in ICC cancer cells and was strongly associated with a poor prognosis. The inhibition of MAL2 effectively suppressed cell proliferation, invasion, and migration. Transcriptomics and metabolomics analyses suggested that MAL2 promoted lipid accumulation in ICC by stabilizing EGFR membrane localization and activated the PI3K/AKT/SREBP-1 axis. Molecular docking and Co-IP proved that MAL2 interacted directly with EGFR. Based on constructed ICC organoids, the downregulation of MAL2 enhanced apoptosis and sensitized ICC cells to cisplatin. Lastly, we conducted a virtual screen to identify sarizotan, a small molecule inhibitor of MAL2, and successfully validated its ability to inhibit MAL2 function. Our findings highlight the tumorigenic role of MAL2 and its involvement in cisplatin sensitivity, suggesting the potential for novel combination therapeutic strategies in ICC.

## Introduction

Intrahepatic cholangiocarcinoma (ICC), trailing only hepatocellular carcinoma, is the second most prevalent form of primary liver cancer, making up 20% of such malignancies, with a marked increase in incidence over the last 4 decades [1]. Current reports indicate that a curative surgical approach is accessible to a mere 30–40% of ICC patients. Regrettably, high recurrence rates mean that survival beyond 5 years is a reality for fewer than one in three patients who undergo potentially curative surgery [2]. The use of cisplatin-based combination therapy has become the standard initial treatment for individuals with advanced or recurrent ICC, although it only marginally extends overall survival (OS) periods [3]. Given these circumstances, it is crucial to investigate the underlying molecular processes involved in ICC with the goal of devising innovative therapeutic approaches.

The unparalleled potential of single-cell RNA sequencing (scRNA-seq) techniques makes it a powerful tool for single-cell transcriptome analysis, especially in identifying molecular signatures implicated in tumor onset and advancement. Such approaches allow for a highly precise evaluation of transcript sequences, paving the way for a more in-depth understanding of the cellular heterogeneity in the tumor microenvironment and the intricate interplays between cells within diverse malignant tissues [4, 5]. Utilizing scRNA-seq in our investigation, we observed that the tetra-transmembrane protein mal, T cell differentiation protein 2 (MAL2) was markedly upregulated in ICC cancer cells, whereas it showed drastically reduced expression in other cellular types, including immune cells. As a member of the MAL protein family, MAL2 is recognized for its role in controlling the hepatic apical cell pathway [6, 7]. Elevated levels of MAL2 expression have been found in various cancers such as breast, hepatocellular, and pancreatic malignancies, often correlating with a grim prognosis for patients [8–10]. Nevertheless, the question of whether MAL2 plays a role in the progression of ICC and the emergence of drug resistance is yet to be answered. Therefore, shedding light on the function of MAL2 could be pivotal in gaining insight into the pathogenesis of ICC and in the discovery of potential novel biomarkers or therapeutic targets.

Our current research indicates that a high level of MAL2 expression is linked to a worse prognosis for patients. Subsequent experiments revealed that by suppressing MAL2, the propensity of the tumor to promote growth is reduced. Critically, we established that the membrane-bound protein MAL2 fosters ICC lipid metabolism through maintaining the membrane positioning of EGFR and the sustained activation of the PI3K/AKT/SREBP-1 pathway, thus reinforcing the malignant phenotype of ICC. Lipids, largely composed of fatty acids and cholesterol, are a diverse and intricate class of molecules vital for cancer development, progression, and persistence [11]. Research has indicated a marked upregulation of crucial genes for lipid metabolism in cholangiocarcinoma (CCA) tissues, correlating significantly with a grim prognosis for patients [12, 13]. To support rapid proliferation and metastasis, CCA cells harness energy through the absorption of extracellular free fatty acids and lipoproteins [14]. Modifications in lipid metabolism are found to be linked with tumor drug resistance [15, 16]. Nonetheless, the mechanisms connecting lipid metabolism in ICC cells with cancer cell growth, metastasis are still shrouded in ambiguity. Furthermore, employing a virtual screen, we present the first evidence of sarizotan, a selective and cell-active inhibitor of MAL2. To our understanding, it represents a distinct variant of MAL2 inhibitor and a potentially effective small molecule in curbing ICC expansion.

## Materials and methods

### Single-cell RNA data analysis

Utilizing data procured from the GEO database (GSE138709), we conducted an examination of the fluctuations in MAL2 mRNA expression within ICC. The selection of individual cells for in-depth analysis was guided by the following prerequisites: the proportion of mitochondria should not exceed 10% of the total unique molecular identifier (UMI) count, and the counts of UMI should fall within the range of 3000–40,000. Subsequent to these steps, the gene expression scale (UMI + 1) was normalized and subjected to a LOG2 transformation.

### Samples collection

From 2020 to 2021, the First Affiliated Hospital of Nanjing Medical University provided treatment for 67 ICC cases, who ranged in age from 34 to 80 years (average age, 60.5 years). These patients underwent their first radical resection for paired ICC tissues and corresponding normal tissues. We excluded individuals who had received radiation or chemotherapy prior to the surgery or those whose clinicopathological data were incomplete. The pathological attributes of ICC were mutually agreed upon by two pathologists. We estimated OS from the time of the first surgery to the date of death from any cause or to the date of the latest follow-up. The study received approval from the Ethical Committee of the First Affiliated Hospital of Nanjing Medical University.

### Cell and cell culture

We procured human intrahepatic bile duct epithelial cell lines along with human ICC cell lines (HUCCT1 and RBE) from the Chinese Academy of Sciences Type Culture Bank. These cells were then grown in DMEM (Gibco, USA) supplemented with 10% fetal bovine serum and 1% penicillin/streptomycin. The culturing of these cells was maintained at a steady temperature of 37 °C with a 5% CO_2_ environment in a temperature-controlled incubator.

### Cell transfection

In human ICC cells (HUCCT1 and RBE), we implemented the construction of MAL2 plasmids and lentiviral packaging (Genechem, China). Using Human shRNA (Genechem, China), the expression of MAL2 was suppressed in these cells. Initial cell cultivation involved seeding 1 × 10^5^ cells in each well of a six-well plate and incubating them with 2 ml of medium for a period of 24 h. After this, we reduced the medium to 1 ml and introduced an adequate quantity of virus and 40 µL of polybrene (Sigma-Aldrich, USA). Subsequent to an incubation period spanning 12–16 h, the cells were placed under normal medium and subjected to puromycin screening. The constructed shRNAs sequences are documented in Table S1. The effectiveness of transfection was monitored via qRT-PCR and western blotting tests. Human-sh1-MAL2 was finally chosen for subsequent experiments. Overexpression trials involved the insertion of MAL2’s cDNA into pcDNA3.1. The transfection of cells with the plasmid was facilitated using Lipofectamine 2000 (Invitrogen, USA), with pcDNA3.1 plasmid vector serving as a control.

### RNA extraction and quantificational real-time polymerase chain reaction (qRT-PCR)

We utilized the FastPure® Cell/Tissue Total RNA Isolation Kit V2 (Vazyme, China) to extract total RNA from cells and samples as per the provided instructions by the manufacturer. By employing a reverse transcription kit (Vazyme, China), the RNA was transcribed in reverse into cDNA. The specific sequences of each primer are presented in Table S1. In order to establish normalized mRNA expression levels, we used GAPDH as a reference.

### Flow cytometry detection and analysis

By using the cell counting method, we determined the overall quantity and intensity of ICC cells. For the staining of cell surfaces, we re-suspended the cells in 2% serum-containing PBS. We then added EGFR antibody (Bioss, China), which is amenable for flow cytometry detection, and the cells were kept on ice for half an hour. Post-incubation, we performed two washes using PBS and then carried out detection via flow cytometry. The resultant data were evaluated employing Flowjo V10 software.

### Cell proliferation assay

Both HUCCT1 and RBE cells were independently plated in 96-well plates and categorized into test and control groups. To plate 1000 cells, we utilized 100 μL of media in each well and incorporated 10 μL of CCK-8 solution (RiboBio, China). We used a microplate reader to measure cell absorbance (OD) at 450 nm at 0, 24, 48, and 72 h of culture following the guidelines provided by the manufacturer (Synergy, USA). For evaluation of cellular proliferation capacity, we performed Cell-Light 5-ethynyl-2′-deoxyuridine (EdU) assays with the EdU DNA Cell Proliferation Kit (RiboBio, China). In a 24-well plate, each well was populated with 50,000 cells. Post-regular culture, cells were exposed to 50 mmol/L EdU solution for a period of 2 h and subsequently fixed with 4% paraformaldehyde. In accordance with the kit’s instructions, we treated the cellular strains with Apollo Dye Solution and DAPI, followed by imaging and enumeration using an Olympus FSX100 microscope (Olympus, Japan).

### Transwell migration and invasion assays

In accordance with the guidelines provided by the manufacturer, we populated the upper chamber with 20,000 HUCCT1 cells and RBE cells, respectively, utilizing 200 μL of serum-free DMEM medium and divided the setup into test and control batches. We utilized Transwell chambers (Corning, USA) loaded with Matrigel mix (BD Biosciences, USA) to evaluate the invasive capabilities of the cells. However, the Matrigel mixture was omitted during the assessment of cellular migratory aptitude. In the lower chamber, we placed 700 μL of DMEM medium imbued with 10% FBS to bind with the chemotactic agent of ICC cells. Following a 24-h incubation period, we removed the upper chamber, aspirated the serum-free medium, and subjected it to a 10-min fixation with 4% paraformaldehyde. Subsequent staining was achieved with crystal violet (Kaigen, China) for 15 min, followed by a wash with PBS. Microscopic imaging was performed on the cellular strains, with counts taken from five distinct areas.

### Wound healing assay

HUCCT1 and RBE cells were cultured in a six-well plate and allowed to grow to confluence. A standard 20 μL pipette tip was used to manually create a linear wound on the merged cell monolayer. Upon the removal of floating cells with a PBS wash, we supplemented the environment with complete medium and proceeded with incubation at 37 °C. Using an inverted microscope, we captured images at 0, 24, and 48 h, with the width of the scratch quantified. All experiments were conducted in sets of three for each group.

### Cell apoptosis assay

We utilized a Cytoflex flow cytometer (Beckman, China) for the identification of cell apoptosis. We adhered to the manufacturer’s instructions to employ the Annexin V-PE/7AAD Apoptosis Detection kit (Vazyme Biotech, China) for the quantification of cell apoptosis. Subsequent data interpretation was performed using the Flowjo V10 software.

### Western blotting

Cell proteins were isolated employing a RIPA buffer, subsequently solubilized with SDS–PAGE, and then migrated onto PVDF membranes. The membranes were incubated with specific primary antibodies at 4 °C overnight. We employed an enhanced chemiluminescence assay (ECL) (Thermo Fisher, USA) for visualizing the antigen–antibody interaction, with the secondary antibody conjugated to peroxidase. The information of antibodies can be found in Table S2.

### RNA sequencing

To scrutinize the impacts of MAL2 suppression on global gene expression, we processed both MAL2 silenced and control HUCCT1 cells with Trizol for lysis, followed by RNA sequencing. The RNA sequencing data were then evaluated by Berry Genomics (Beijing, China).

### Co-immunoprecipitation (Co-IP) assay

HEK293T cells were introduced with the specified plasmids and allowed to incubate for a period of 24 h. Following this, the cells underwent lysis in an IP lysis buffer, which had protease and phosphatase inhibitor tablets, for half an hour. Post centrifugation at 12,000 rpm at a temperature of 4 °C, the supernatant, which contained the protein, was subjected to overnight incubation with protein G agarose beads (Bestchrom, China) and specific antibodies at 4 °C. Subsequently, the beads were thoroughly rinsed three times with buffers having 300 and 150 mM NaCl and then were treated with a 2 × SDS loading buffer at 4 °C before executing the immunoblotting process. The information of antibodies can be found in Table S2.

### Immunofluorescence and immunohistochemistry

Immunofluorescent cells were fixed with 4% paraformaldehyde (Generalbiol, China) for a period of 20 min under ambient conditions, followed by cell permeabilization for 10 min using 0.05% Triton X-100 (Sigma-Aldrich, USA). The specimens were then blocked using PBS that contained 2% BSA and left to stand for 60 min at room temperature, following which they were incubated at 4 °C throughout the night with specific antibodies. Further, these samples were incubated with Alexa Fluor secondary antibodies for 60 min at room temperature. Nuclei were counterstained with Hoechst 33342 (Sigma-Aldrich, USA) and visuals were captured via laser scanning confocal microscopy. In terms of immunohistochemistry studies, the samples were kept overnight at 4 °C with specific antibodies and immune responses were detected the following day using 3′-diaminobenzidine (Generalbiol, China), in line with the supplier’s guidelines. The information of antibodies can be found in Table S2.

### Palmitic acid (PA) concentration detection

The evaluation of PA was conducted utilizing a PA ELISA kit commercially procured from Meimian Biotechnology, China. The PA assays were in compliance with the protocols stipulated by the kit manufacturer. Subsequent analysis was executed through a microplate reader, quantifying the sample’s absorbance (OD) at a wavelength of 450 nm.

### Metabolomics sequencing and detection

A concoction of methanol, acetonitrile, and water was prepared in a 2:2:1 proportion. After cooling, 1 ml of this blend was sonicated for 60 min in ice water. The sample then underwent an hour-long incubation at −20 °C. Centrifugation at 14,000 × *g* at a temperature of 4 °C was performed for 20 min to facilitate LC–MS analysis. The evaluation of metabolomics utilized a UPLC-ESI-Q-Orbitrap-MS system (UHPLC, Shimadzu Nexera X2 LC-30AD, Shimadzu, Japan), complemented by Q-Exactive Plus (Thermo Scientific, USA). Data were obtained via electrospray ionization in both positive and negative modes. Quality control (QC) samples were made by combining all aliquots, followed by data normalization. Every six samples saw the injection of QC and blank samples (75% acetonitrile in water).

### Metabolome sequencing analysis

MS-DIAL was employed to analyze the raw MS data. Comparisons were drawn between public databases and our standard metabolite library using MS/MS data. Permutation tests were used to fit all evaluation models. The use of OPLS-DA facilitated the identification of metabolites via variable importance for the projection (VIP) analysis. VIPs with a mean value exceeding 1 were deemed substantial, with higher values indicating greater significance; this became the benchmark for biomarker selection. Univariate analysis level used statistical significance thresholds and two-tailed Student’s *t*-tests (*P* values) for metabolite assessment. In cases of multiple group analyses, ANOVA was used to compute *p* values. VIP values above 1.0 and *P* values below 0.05 were taken as statistically significant. The R package was used for the cluster analysis of the recognized differential metabolites. The KEGG database (http://www.kegg.jp) undertook KEGG pathway analysis of the differential metabolite data. KEGG enrichment analysis was conducted using multiple tests, which included Fisher’s exact test and FDR correction. KEGG pathways enriched were considered statistically significant if *P* values were below 0.05.

### Virtual screening and molecular docking

AlphaFold was employed for estimating potential active sites, taking into account the structure of MAL2 (ID: AF-Q969L2-F1). A visual screening was performed on two commercial chemical libraries comprising over 67,000 compounds, leveraging the grid-based ligand docking capabilities of GLIDE software (Schrödinger Maestro 11.4) to pinpoint the anticipated locations. The highest five scoring compounds, procured from MedChemExpress, were designated for testing.

A docking study was carried out with HDOCK to investigate the binding modes between EGFR and MAL2. EGFR (PDB ID: 1MOX) was retrieved from the PBD database, and MAL2 was obtained from the UniProt database. The protein structures were subsequently examined using Pymol 2.3.0 to evaluate the interaction patterns reflected in the docking results.

### Microscale thermophoresis (MST)

In the course of our experimental work, we utilized the Monolith NT.115 (NanoTemper Technologies GmbH, Germany) for Microscale thermophoresis (MST) adhering to the specified protocols provided by the manufacturer. We sourced fluorescently labeled MAL2 from lysates of HEK293T cells, specifically engineered to express GFP-MAL2. For the purpose of studying sarizotan’s interaction with MAL2, GFP-MAL2 plasmids were transfected into HEK293T cells, which were subsequently lysed a day post-transfection. Post-transfection, we proceeded to incorporate a titration series of sarizotan (MedChemExpress, China), which varied from 0 to 1 mM, with our diluted cell lysate sample. The lysates underwent dilution at a ratio of 1.5:1 with MST buffer (containing 10 mM Na–phosphate buffer at pH 7.4, 1 mM MgCl_2_, 3 mM KCl, 150 mM NaCl, and 0.05% Tween-20) to achieve an optimal level of fluorescence. Lysates from untransfected HEK293T cells acted as a control to examine background fluorescence, which was found to be negligible in our MST system. We carried out our measurements within a high-quality coated capillary (provided by NanoTemper Technologies GmbH, Germany) utilizing a 470 nm LED light source. The conditions were set at full infrared laser power and a maintained temperature of 25 °C. We employed the MO Affinity Analysis v2.1.3 software (NanoTemper Technologies GmbH, Germany) to normalize the fluorescence signal and apply the Hill equation fitting.

### In vivo nude mouse model

We procured 6-week-old BALB/c nude mice from the Model Animal Research Center at Nanjing Medical University. Each specimen underwent a thorough health check before tumor implantation to guarantee their disease-free status. We operated within the framework approved by the Animal Care Committee of Nanjing Medical University. For our study, each experimental group comprised five nude mice, which were subcutaneously administered 1 × 10^6^ transfected HUCCT1 cells. Following the inoculation of tumor cells via subcutaneous injection after 7 days, the mice were administered intraperitoneal injections of either cisplatin (which was dissolved in DMF at a dosage of 4 mg/kg on a weekly basis) or the vehicle (DMF). With regard to the sarizotan treatment trials, 7 days post the subcutaneous administration of 1 × 10^6^ HUCCT1 cells, the mice received intraperitoneal injections every 3 days of either sarizotan (dissolved in DMSO at a concentration of 5 mg/kg) or the vehicle (DMSO). At the conclusion of the 4-week study period, we euthanized the mice. The tumors were subsequently extracted, and the volume was computed using the formula: volume (mm^3^) = 0.5 × width^2^ × length.

For liver orthotopic-implantation models, 3 × 10^6^ HUCCT1 cells were injected into the liver of each mouse. Seven days after implantation, orthotopic transplanted nude mice were randomly assigned to four groups: Control, Sarizotan, Cisplatin, and a combination of Sarizotan and Cisplatin. For sarizotan-treated nude mice, 4 mg/kg of sarizotan was injected intraperitoneally every 3 days, and for cisplatin-treated mice, 5 mg/kg of cisplatin was administered by intraperitoneal injection once a week. All nude mice were executed after 3 weeks and liver tumor tissue was harvested.

### Isolation and culture of human ICC organoids

We meticulously dissected small sections of the cancerous tissue, each measuring ~1 cm^2^. These were rinsed in Advanced DMEM/F12 medium (sourced from GIBCO, USA) until no particulate matter could be detected in the supernatant. To achieve enzymatic disintegration of these fragments, we employed a concoction of 1.5 mg/mL collagenase (Gibco, USA) and 20 µg/mL hyaluronidase (procured from Sigma-Aldrich, USA). The tissues were then incubated in 10 mL of the aforementioned DMEM/F12 medium at 37 °C with constant agitation for a period of 1 h. The resulting cellular suspension was sieved using a 70 µm strainer. Once the cells had been thoroughly washed in the Advanced DMEM/F12 medium, they were seeded into a substrate of growth factor reduced Matrigel (Corning, USA). The final medium composition included HEPES, Glutamax, Penicillin, Streptomycin, B27, n-Acetylcysteine, EGF, R-spondin1, Noggin, Wnt, FGF10, Gastrin, TGF-inhibitor, and RHOK-inhibitor. For maintenance, the organoids were split and subcultured on a weekly basis at ratios varying between 1:3 and 1:10.

### Nile red staining

The ICC cell lines, namely HUCCT1 and RBE, were enumerated and subsequently seeded into six-well plates at a density of 1 × 10^5^ cells/well. This procedure was conducted 48 h post-transfection. Staining procedures were initiated 24 h thereafter with Nile red, a fluorescent dye obtained from Solarbio Life Sciences, China. Adherence to the manufacturer’s staining guidelines was strictly maintained to ensure consistency. Finally, an inverted fluorescent microscope from Nikon, Japan was employed to capture the micrographs.

### Reagents

BP (palmitoylation inhibitor) was bought from Sigma-Aldrich, USA. Fatostatin (SREBP-1 inhibitor), EGF, CHX, AG1478 (EGFR inhibitor), Sarizotan, and PA were bought from MedChemExpress, China.

### Statistical analysis

Data were shown as the mean ± standard deviation. GraphPad Prism 8.0 was utilized as the primary tool for performing the bulk of the analytical tasks in this study, where a *p* value of less than or equal to 0.05 was considered indicative of statistical significance. Student’s *t*-tests or ANOVA were deployed for comparative analyses across the various groups within the study. OS was analyzed using the Kaplan–Meier method and significance was determined by log-rank test. The correlation between MAL2 expression and clinicopathological characteristics was assessed using the *χ*^2^ test. Cox proportional risk regression model was used to determine independent prognostic factors.

## Results

### MAL2 expression is upregulated in ICC tissues based on scRNA-seq results and correlated with worse clinical outcomes

**Fig. 1:**
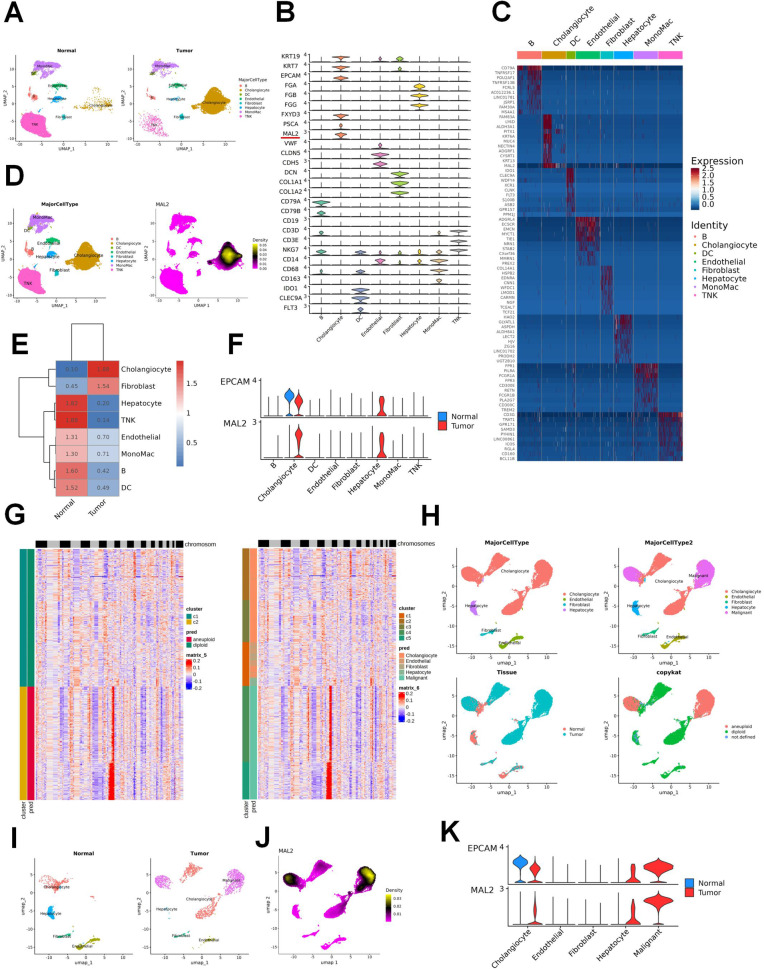
Outlines the acquisition of scRNA-seq profiles for various ICC samples and subsequent data generation. **A** A UMAP visualization representing eight unique cell types identified within normal and tumoral tissues. **B** Utilization of a violin plot to depict the distinct cellular populations and their respective differential markers. **C** Heat maps presenting the differentiation of cell populations and their distinguishing markers. **D** Another UMAP representation showcasing the heterogeneity in MAL2 expression across various clusters. **E** The heat maps depict differential expressions within varied cell populations derived from disparate samples. **F** The violin plot portrays the diversity in MAL2 expression within differing clusters and tissues. **G** Clustering heatmap of CopyKAT-estimated copy number profiles of scRNA-seq. **H** UAMP visualization of malignant cholangiocyte clusters identified on the basis of CNV. **I** A UMAP visualization representing five unique cell types identified within normal and tumoral tissues. **J** UMAP representation showcasing the heterogeneity in MAL2 expression across various clusters. **K** The violin plot portrays the diversity in MAL2 expression within differing clusters and tissues.

The GEO database (GSE138709) was employed to examine the fluctuation of MAL2 mRNA expression in ICC. From the UMAP plot and heatmap, distinct gene markers helped classify GSE138709 findings into eight cell clusters, namely, cholangiocytes, fibroblast, hepatocyte, TNK, endothelial, MonoMac, B lymphocytes, and dendritic cells (DC), as showcased in Fig. 1A–C and Fig. S1A. A specific expression of CD79A was found in the B cluster, as depicted in Fig. 1B, C and Fig. S1A. Interestingly, cholangiocytes and fibroblasts exhibited more robust expression in tumor tissues than in regular tissues. Conversely, tumor tissues displayed a decrease in the abundance of hepatocytes, TNK, B cells, and DCs when compared to normal tissue samples, as seen in Fig. 1E and Fig. S1B. To our surprise, MAL2 was found to be significantly abundant in cholangiocytes (Fig. 1D and Fig. S1C). The expression levels of MAL2 were more prevalent in tumor tissues than in normal ones (Fig. 1F). Chromosome copy number variation (CNV) of different cells in ICC tissue was analyzed based on CopyKAT, a computational tool. We found that non-immune cells such as endothelial cells were normal diploid, and classified cholangiocytes in ICC into normal cholangiocytes and malignant cholangiocytes based on whether or not there was a change in CNV (Fig. 1G, H). Malignant cholangiocytes were almost distributed in ICC, and MAL2 was expressed mainly in malignant cholangiocytes in ICC (Fig. 1I–K). These revelations point toward the crucial role MAL2 might play in ICC cancer cells.

To further assess the expression level of MAL2, we applied the immunohistochemistry assay to 67 paraffin-embedded specimens obtained from ICC patients, revealing that ICC tissues indeed possess higher levels of MAL2 protein than adjacent paracancerous tissues, a finding that echoes the results from the TCGA (Fig. S2A, B). Representative MAL2 high or low expression immunohistochemistry is shown in Fig. S2C. Increased MAL2 mRNA and protein expression levels in ICC cell lines compared to normal bile duct epithelial cells (Fig. S2D, E). Clinical-pathological features suggested that MAL2 expression has a significant correlation with TNM classification and lymph node metastasis, although no significant correlation with age, gender, or vascular invasion (Table S3). The Kaplan–Meier survival curve showed a reduced OS rate for patients with ICC who had high MAL2 expression or lymph node metastases (Fig. S2F). Univariate and multivariate analysis of Cox proportional risk analysis showed high levels of MAL2 with independent predictors of OS after surgery in patients with ICC (Fig. S2G). These findings highlight that MAL2 is significantly upregulated in ICC, suggesting a grim prognosis for ICC patients.

### MAL2 promotes ICC proliferation, migration, and invasion

**Fig. 2:**
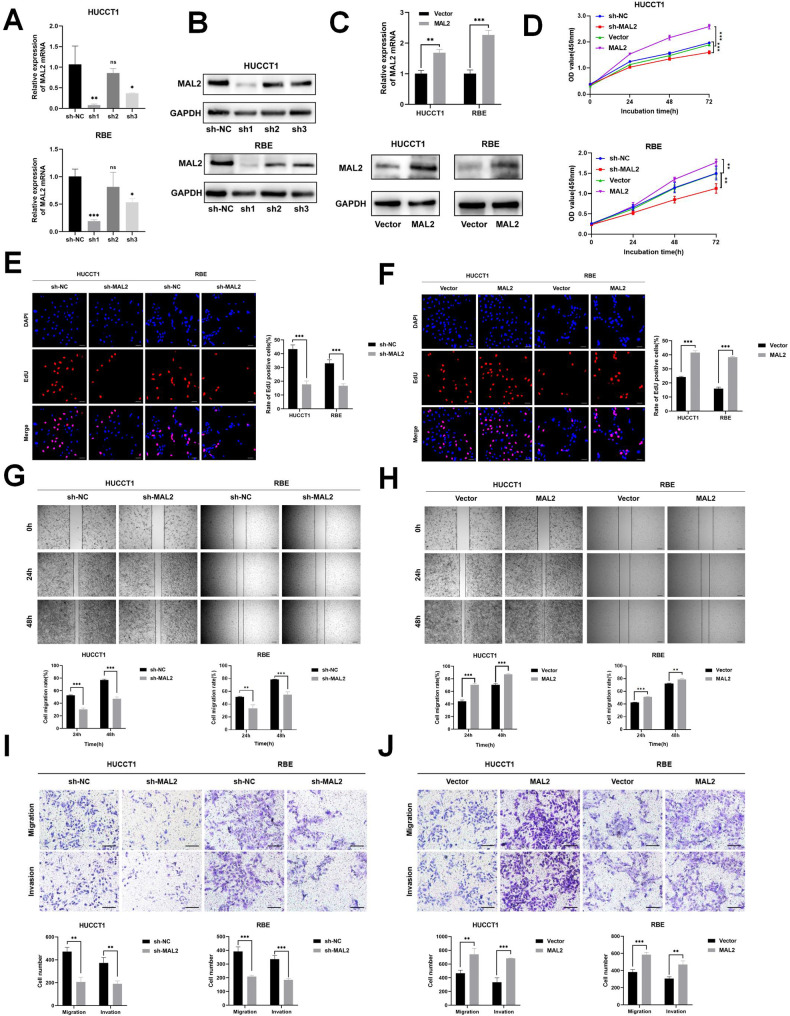
The substantial impact of MAL2 knockdown in hindering ICC cell proliferation, invasion, and migration. **A** Comparative analysis of MAL2’s mRNA expression levels in HUCCT1 and RBE cells following transfection with either sh-NC (negative control) or sh-MAL2 (sh1, sh2, and sh3). **B** Quantification of MAL2 protein expression in HUCCT1 and RBE cells post-transfection with sh-NC or sh-MAL2 (sh1, sh2, and sh3) using western blotting technique. **C** Relative mRNA and protein expression of MAL2 in HUCCT1 and RBE cells transfected with control (negative control) or MAL2 vector. **D** Growth kinetics of HUCCT1 and RBE cells, post-transfection with sh-NC, sh-MAL2, control or MAL2 vector, was charted based on the CCK-8 assay data at various time points – 0, 24, 48, and 72 h. **E**, **F** Cell proliferation in ICC cells post-transfection with sh-NC, sh-MAL2, control, or MAL2 vector was evaluated using an EdU assay; the scale bar represents 50 μm. **G**, **H** A wound healing assay assessed the migratory capacity of HUCCT1 and RBE cells post-transfection with sh-NC, sh-MAL2, control, or MAL2 vector; the scale bar represents 50 μm. **I**, **J** Invasion potential of HUCCT1 and RBE cells post-transfection with sh-NC, sh-MAL2, control, or MAL2 vector was investigated using a Transwell invasion assay; the scale bar stands for 200 μm. *P < 0.05; **P < 0.01; ***P < 0.001.

In the proceeding phase of our investigation, we formulated three shRNA structures (referred to as sh1, sh2, and sh3) aiming to silence MAL2 in HUCCT1 and RBE cells. A combined examination through qRT-PCR and western blotting revealed that sh1 yielded a considerable reduction in MAL2 expression, as evidenced in Fig. 2A, B. We also attempted to generate MAL2-overexpressing ICC cell lines. This was accomplished by transfecting HUCCT1 and RBE cells with plasmids expressing MAL2, as shown in Fig. 2C. Assessments using the CCK-8 and EdU tests brought to light that the proliferation of HUCCT1 and RBE cells showed an upswing when MAL2 was overexpressed, compared to the control group. Conversely, when MAL2 expression was suppressed, the proliferation of these cells witnessed a decline, as visualized in Fig. 2D–F. Wound healing experiments demonstrated that MAL2 downregulation in HUCCT1 and RBE resulted in a substantial decrease in cell migration. However, an uptick in cell migration was noticed when MAL2 was overexpressed in the same cell lines (Fig. 2G, H). Transwell experiments further revealed that a reduction in MAL2 levels curtailed the migration and invasion capabilities of ICC cells. Conversely, a pronounced increase in these abilities was observed when MAL2 was overexpressed (Fig. 2I, J). In summary, these collective findings suggest that MAL2 could potentially enhance the proliferative and motility characteristics of ICC cells.

### Metabonomics and RNA sequencing analysis of downregulating MAL2 in ICC cells

**Fig. 3:**
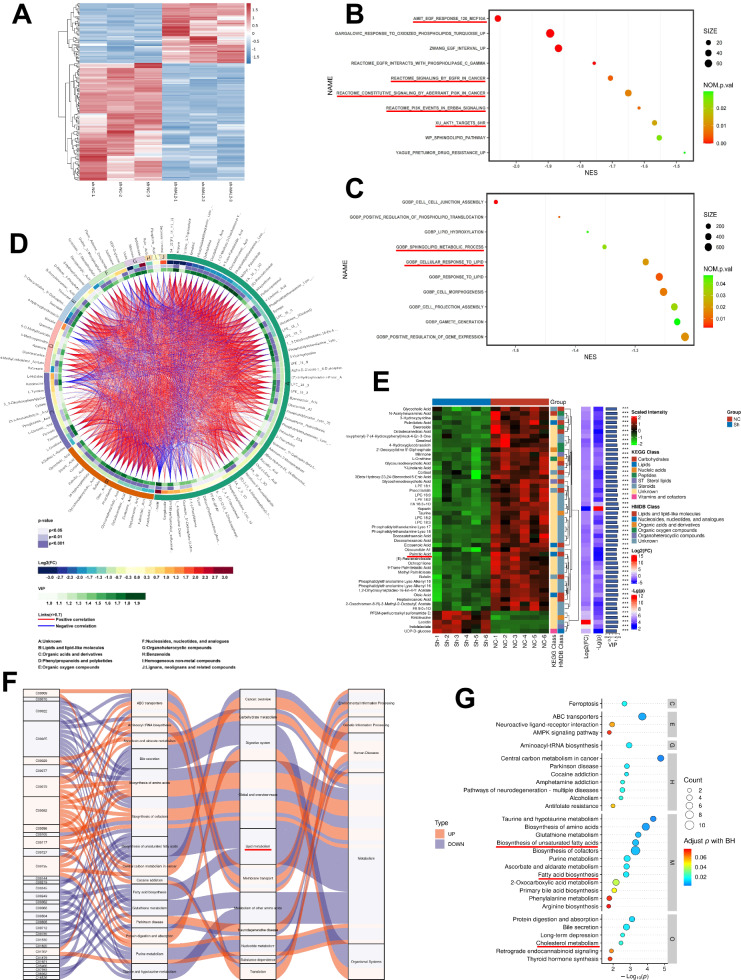
Transcriptomic and metabolomic exploration upon MAL2 suppression in ICC cells. **A** A heatmap displays shifts in gene expression subsequent to MAL2 silencing in ICC cells. **B**, **C** Pathway enrichment of KEGG and GO among the diverse genes expressed. **D** A Circos plot illustrating the associations among various disparate metabolites. **E** Classification and tallying of distinctive metabolites in every control group, based on their structure and function, are demonstrated, presenting classification data from both KEGG and HMDB databases. **F** A Sankey diagram showing the trajectories of downregulated metabolite data flow across multiple pathways. **G** KEGG pathway investigation pinpointing the key pathways where these altered metabolites are heavily represented.

Our previously mentioned in vitro experiments established that MAL2 promotes the invasive, migratory, and proliferative properties of ICC. To delve deeper into the potential molecular mechanisms through which MAL2 may facilitate ICC progression, we employed RNA sequencing on control HUCCT1 cells and HUCCT1 cells where MAL2 was knocked down. Differentially expressed genes can be seen in the heat maps and volcano plots (Fig. 3A and Fig. S3A). Upon observing the sh-MAL2 group, it was found that a total of 96 mRNAs were significantly reduced, whereas 49 mRNAs showed considerable upregulation (Fig. S3B). KEGG analysis of the results revealed that a large proportion of the differentially downregulated genes were clustered in the PI3K/AKT pathway, EGFR signaling pathway, and EGF responses, as depicted in Fig. 3B. From the gene ontology (GO) analysis, the most prevalent activities associated with these genes involve biological processes such as sphingolipid metabolic process and cellular response to lipid, as well as molecular functions including cell junction and signaling activity (Fig. 3C). Additionally, analysis of the differentially upregulated mRNAs unveiled a notable enrichment in pathways related to aging and tumor formation (Fig. S3C, D). The gene set enrichment analysis also suggested a potential correlation with the regulation of activity in the epidermal growth factor-activated receptor, implicating a diverse set of genes (Fig. S3E).

Metabolomics technologies have proven invaluable in facilitating a detailed analysis of metabolites and conducting thorough and precise investigations of cytokines and associated signaling pathways [17]. In our current research, metabolomics sequencing was employed on HUCCT1 cells from both the sh-NC and sh-MAL2 groups. The correlation among multiple differential metabolites was chiefly represented in the cycle diagram (Fig. 3D). Volcano plots were utilized to exhibit differential metabolites (Fig. S4A, B), with the ratio of diverse types of metabolites depicted in Fig. S4C. Classification and tallying of the varying metabolites within each comparison group were conducted according to the structural and functional characteristics of the metabolites, with KEGG and HMDB databases providing the results of substance classification (Fig. 3E). Employing Sankey plots for a visual examination of the data flow trends among differential metabolites and several pathways revealed that the largest accumulation of differential metabolites in the far-right hierarchy was found in metabolism. Meanwhile, the highest concentration within metabolic pathways was observed in the global and overview representations of lipid metabolism (Fig. 3F). KEGG pathway analysis further showed that these differential metabolites were predominantly centered around the biosynthesis of unsaturated fatty acid, fatty acid biosynthesis, and cholesterol metabolism, among others (Fig. 3G and Fig. S4D). Collectively, these findings imply a significant association between MAL2 and lipid metabolism.

### MAL2 mediates ICC cells lipid accumulation through regulation of the PI3K/AKT/SREBP-1 pathway

**Fig. 4:**
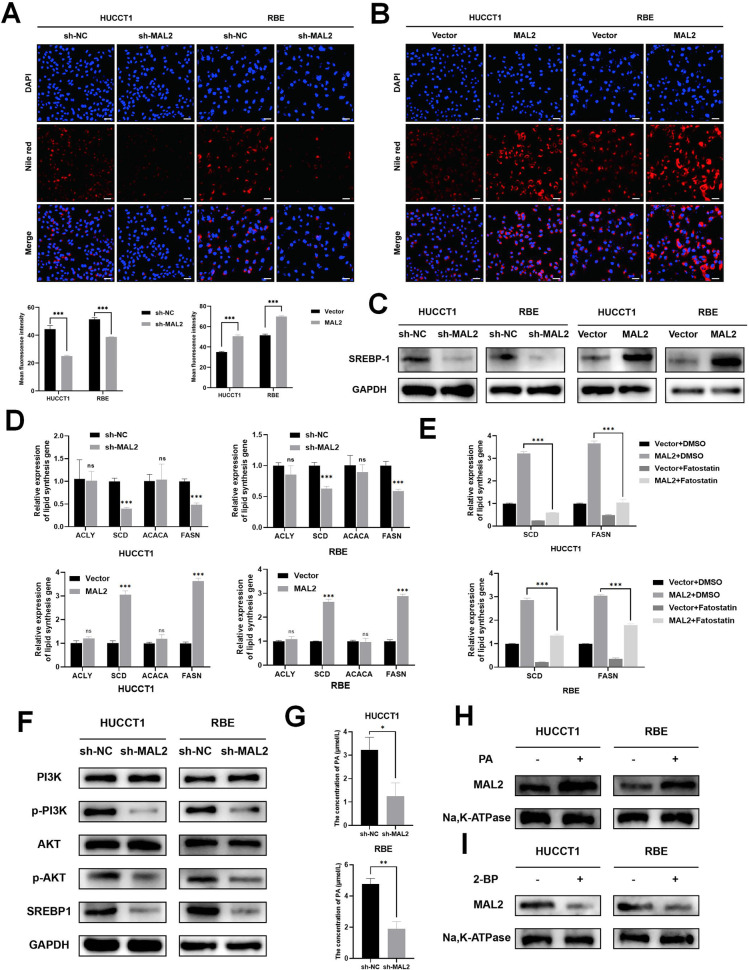
MAL2 enhancement of cellular lipid storage in HUCCT1 and RBE cells through the activation of PI3K/AKT signal pathway. The baseline LDs content in sh-NC or sh-MAL2-introduced (**A**), and control or MAL2 vector-introduced (**B**) HUCCT1 and RBE cells was evaluated using Nile red staining, scale bar, 50 μm. This was followed by quantifying the mean fluorescence intensity of Nile red staining for each cell line. **C** Western blotting methodology was implemented to investigate the expression of SREBP-1 post-MAL2 suppression and overexpression, using GAPDH as a loading standard. **D** In HUCCT1 and RBE cells, post-MAL2 suppression and overexpression, the relative mRNA expression of lipid synthesis genes was probed through qRT-PCR. **E** In HUCCT1 and RBE cells, post-Fatostain treatment (20 µM, 48 h), the relative mRNA expression of SCD and FASN was probed through qRT-PCR. **F** The impact of MAL2 on the PI3K/AKT signal pathway in sh-NC or sh-MAL2-transfected HUCCT1 and RBE cells was explored using western blotting. **G** Results of determining the PA concentration in each sample group. **H**, **I** ICC cell line membrane expression of MAL2, stimulated by PA or 2-BP, was discerned via western blotting. *P < 0.05; **P < 0.01; ***P < 0.001.

Building on the findings from the preceding RNA sequencing and metabolomics investigations, our research aim was to delineate the mechanism by which MAL2 promotes tumor cell growth through lipid metabolic pathways. Past studies indicate that when cells experience an excess of lipids, the endoplasmic reticulum proceeds to convert these lipids into triglycerides and cholesterol esters, which subsequently form into lipid droplets (LDs) [18]. Among organelles, LDs are characterized by their distinctive architecture, composed of a dense hydrophobic core of neutral lipids enveloped by a single phospholipid layer. This layer is adorned with a plethora of proteins. Cancer cells often exploit LDs to ensure energy production and thereby foster tumor growth [19]. Nile red staining serves as an indicator of LD content. In this study, we observed that the intensity of Nile red staining diminished with MAL2 knockdown, whereas MAL2 overexpression led to an intensification of Nile red staining in both HUCCT1 and RBE cell lines (Fig. 4A, B). The aforementioned observations strongly support the notion that MAL2 deletion reduces LD quantities within ICC cells.

In a bid to gain a deeper understanding of MAL2’s regulation of lipid metabolism, we further scrutinized alterations at the mRNA and protein levels of several crucial transcription factors and metabolic enzymes involved in lipid production in ICC cells. One such transcription factor, sterol regulatory element binding protein-1 (SREBP-1), is known to orchestrate lipid metabolism [20]. Our research unveiled that the inhibition of MAL2 expression resulted in blocking SREBP-1 from expressing itself at the protein level. However, increased levels of SREBP-1 protein was noticed when MAL2 was overexpressed in the same cell lines (Fig. 4C). Activated SREBP-1 moving to the nucleus triggers the transcription of critical enzymes that encode the adipogenic pathway, such as ATP citrate lyase (ACLY), acetyl CoA carboxylase, fatty acid synthase (FASN), and stearoyl-CoA desaturase (SCD) [21]. As per our qRT-PCR data, SCD and FASN mRNA levels in ICC cells were significantly reduced by MAL2 knockdown, while MAL2 overexpression had the inverse effect of MAL2 knockdown (Fig. 4D). To further clarify whether SREBP-1 is involved in MAL2-mediated regulation of SCD and FASN transcript levels, we performed rescue experiments using Fatostatin, an activation inhibitor of SREBP-1, in MAL2-overexpressing ICC cell lines. The results showed that Fatostatin reversed the MAL2-mediated increase in SCD and FASN transcript levels (Fig. 4E). These observations suggest that MAL2 activates SREBP-1 in ICC, thereby enhancing the transcription of SCD and FASN and subsequently fostering fatty acid synthesis. Research showed that the PI3K/AKT oncogenic signaling pathway in cancer stabilizes and activates SREBP-1 [22]. Considering the RNA sequencing pathway enrichment data previously mentioned, we hypothesized that MAL2 triggers SREBP-1 via the PI3K/AKT pathway. To confirm this, we examined the protein levels of PI3K, p-PI3K, AKT, and p-AKT in ICC cells. The results indicated that MAL2 knockdown dramatically reduced the protein levels of p-PI3K and p-AKT (Fig. 4F). Consequently, the above findings demonstrate that MAL2 escalates SCD and FASN transcriptional levels, stimulating ICC fatty acid synthesis via the PI3K/AKT/SREBP-1 pathway.

In the course of our metabolomics investigation, we unexpectedly observed a significant decline in PA within the group where MAL2 was knocked down (Fig. 3E). PA, a saturated fatty acid with a length of 16 carbons, has been found to function as a signaling molecule on a molecular scale, influencing the progression and evolution of numerous diseases [23]. Through palmitoylation modifications, PA can increase the hydrophobic nature of proteins, thereby enhancing their binding to the membrane [24, 25]. Consequently, we formulated the hypothesis that in ICC cells, PA could be facilitating the membrane localization of MAL2. In an attempt to validate our hypothesis, we carried out ELISA experiments, which revealed a drop in PA levels following the knockdown of MAL2 in HUCCT1 and RBE cells (Fig. 4G). Additionally, the membrane expression of MAL2 experienced an uptick post-stimulation with PA in cultured ICC cell lines (Fig. 4H). Furthermore, the introduction of the palmitoylation inhibitor 2-BP led to a marked decrease in the membrane expression of MAL2 (Fig. 4I). In addition, We found that knockdown of MAL2 inhibited PA-induced promotion of lipid accumulation, proliferation, migration, and invasion in ICC cells (Fig. S5A-C).

### MAL2 interacts with EGFR to block EGF-induced EGFR internalization and activate lipid accumulation

**Fig. 5:**
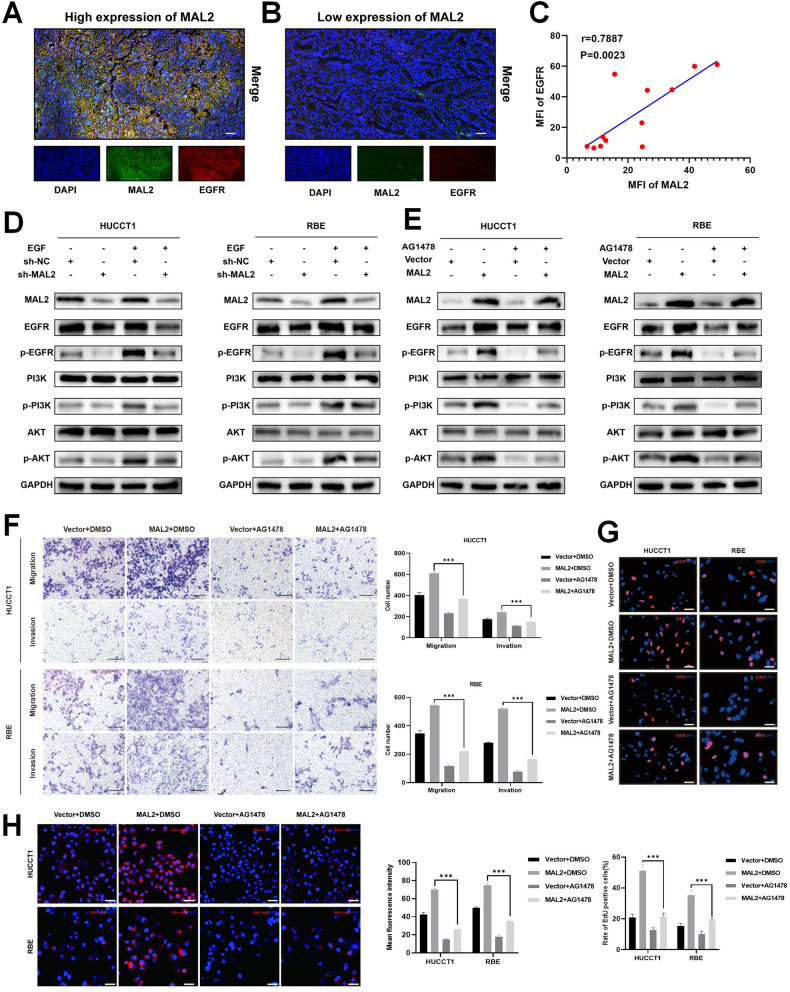
EGFR is involved in MAL2-mediated activation of the PI3K/AKT signaling axis. **A**, **B** Utilizing immunofluorescence, the expression of MAL2 and EGFR in ICC tissues from patients exhibiting high or low MAL2 expression was assessed, scale bar, 50 μm. **C** The correlation between MAL2 and EGFR was analyzed based on mean fluorescence intensity (MFI). **D** The impact of MAL2 on the EGFR/PI3K/AKT signal pathway in sh-NC or sh-MAL2-transfected HUCCT1 and RBE cells post EGF treatment (50 ng/ml, 30 min) was explored using western blotting. **E** The impact of MAL2 on the EGFR/PI3K/AKT signal pathway in vector or MAL2-transferred HUCCT1 and RBE cells post AG1478 treatment (10 μM, 24 h) was explored using western blotting. **F** Invasion potential of HUCCT1 and RBE cells transfected with control or MAL2 vector post AG1478 treatment (10 μM, 24 h) was investigated using a Transwell invasion assay; the scale bar stands for 200 μm. **G** Cell proliferation in ICC cells transfected with control or MAL2 vector post AG1478 treatment (10 μM, 24 h) was evaluated using an EdU assay; the scale bar represents 50 μm. **H** The baseline LDs content in control or MAL2 vector-introduced HUCCT1 and RBE cells post AG1478 treatment (10 μM, 24 h) was evaluated using Nile red staining, scale bar, 50 μm. This was followed by quantifying the mean fluorescence intensity of Nile red staining for each cell line. ***P < 0.001.

The process by which MAL2 activates the PI3K/AKT pathway has not been explicitly defined yet. As per our GO analysis, we discovered that the EGFR pathway was markedly implicated. Notably, EGFR has a substantial association with the development of ICC, with EGFR overexpression noted in 10–32% of ICC patients, adversely affecting their OS [26]. Prior studies indicate that in breast cancer cells, lipid raft formation mediated by MAL2 leads to an enhancement of HER2 signaling pathways and the retention of HER2 at the plasma membrane [27]. Given the reported activation of the PI3K/AKT pathway by EGFR [28], our hypothesis is that EGFR membrane localization could be mediated by MAL2 to activate PI3K. In order to explore the role of MAL2 in EGFR expression within ICC, we gathered 12 ICC samples and used immunofluorescence to analyze EGFR and MAL2 expression. Interestingly, a positive correlation was found between EGFR and MAL2 expression (*r* = 0.7887, *P* = 0.0023) (Fig. 5A–C). Moreover, EGF stimulation induced a significantly increase in the phosphorylation levels of EGFR protein in ICC cell lines reversed the inhibition of the PI3K/AKT pathway induced by MAL2 knockdown, further clarifying the involvement of EGFR in the regulation of the PI3K/AKT/SREBP-1 pathway by MAL2 (Fig. 5D). To further clarify the function of EGFR in MAL2-mediated regulation of lipid metabolism and pro-tumorigenic effects, we performed rescue experiments using the tyrosinase inhibitor AG1478. The results showed that AG1478 reversed the MAL2-mediated increase in phosphorylated EGFR, PI3K, and AKT levels (Fig. 5E). In addition, AG1478 hindered the effects of MAL2 overexpression on the proliferation, migration, and invasion of ICC cells (Fig. 5F, G). Nile red staining assay showed that inhibition of EGFR phosphorylation in MAL2 overexpression ICC cell lines reduced cellular lipid accumulation (Fig. 5H). Collectively, these results suggest that EGFR is involved in MAL2-mediated regulation of lipid metabolism and cellular malignant progression.

**Fig. 6:**
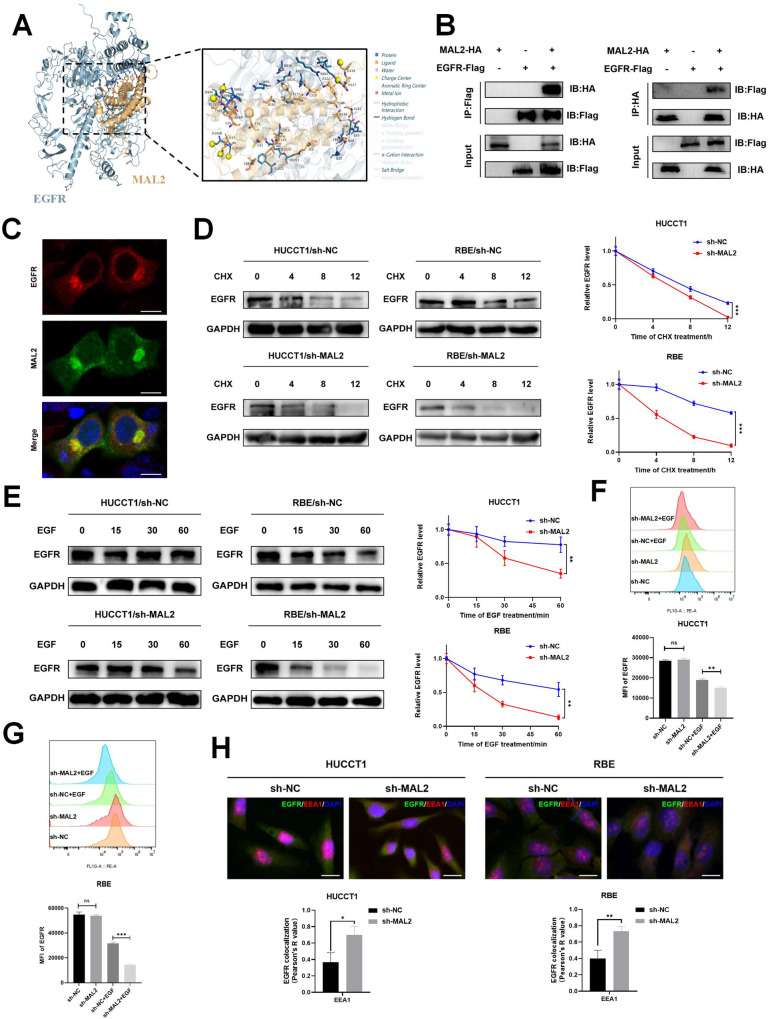
Interaction of MAL2 and EGFR inhibits the degradation of EGFR through endocytosis. **A** Postulated modes of binding between MAL2 and EGFR. **B** Co-IP analysis was conducted to represent the association between MAL2 and EGFR in HEK293T cells transfected with HA-tagged MAL2 and flag-tagged EGFR. **C** Immunofluorescence was used to validate the binding interaction between MAL2 and EGFR, scale bar, 10 μm. **D** ICC cells from different groups treated with CHX (20 μg/ml). Cells were then harvested at specified time frames (0, 4, 8, 12 h) for subsequent western blotting. **E** HUCCT1 and RBE cell lines from sh-NC and sh-MAL2 groups were serum-starved for 24 h. After stimulation with EGF (50 ng/ml) at the indicated time intervals (min), EGFR expression levels were detected by western blot. **F**, **G** HUCCT1 and RBE cell lines from sh-NC and sh-MAL2 groups were serum-starved for 24 h. Exemplary flow cytometry analysis and mean fluorescence intensity of EGFR in HUCCT1 and RBE cells, following treatment with EGF (50 ng/ml, 30 min). **H** The extent of EGFR and lysosomal marker EEA1 co-localization in control and knockdown ICC cells with MAL2 was evaluated via immunofluorescence assay post EGF stimulation (50 ng/ml,15 min), scale bar, 20 μm. *P < 0.05; **P < 0.01; ***P < 0.001.

To clarify whether MAL2 interacts with EGFR, we performed molecular docking of EGFR and MAL2 using Hdock, resulting in a binding energy of −323.16 kcal/mol. The binding interfaces of the protein–protein complexes were comprehensively characterized and systematically analyzed, and interaction-related details were supplemented by PyMOL to form a 3D interaction map. A total of 20 pairs of hydrophobic interactions, 10 pairs of hydrogen bonds, 3 pairs of salt bridges, and 1 pair of π–cation interactions exist between the two pairs of proteins (Fig. 6A). Additionally, we conducted Co-IP experiments to ascertain whether there was a physical interaction between MAL2 and EGFR. Our Co-IP findings revealed an interaction between MAL2 and EGFR (Fig. 6B), and the results from immunofluorescence staining showed that EGFR co-localized with MAL2 at the membrane (Fig. 6C). Next, we examined EGFR protein stability in the HUCCT1/sh-MAL2 and RBE/sh-MAL2 cell lines. We found that the half-life of the EGFR protein significantly declined with MAL2 knockdown, relative to the control. This implies that MAL2 may deter the degradation of EGFR in ICC cells (Fig. 6D).

EGF-induced EGFR internalization and subsequent lysosomal degradation play a critical role in EGFR-related signaling mechanisms [29]. It has been proposed that impaired EGFR endocytosis may drive tumorigenesis by activating several signaling pathways, including the PI3K/AKT pathway, potentially leading to cancer development [30]. To confirm the role of MAL2 in ligand EGF-induced EGFR lysosomal degradation, we treated ICC cells with EGF and found that MAL2 deletion promoted EGF-induced EGFR degradation in HUCCT1 versus RBE cells as compared to the control (Fig. 6E). In an effort to understand how MAL2 governs EGFR activation and degradation, we evaluated EGFR levels on the cell surface of both control and EGF-stimulated cells via fluorescence-activated cell sorting (FACS). The aim was to investigate whether MAL2 could influence the dynamics of EGFR internalization. After EGF stimulation, the group with sh-MAL2 demonstrated faster EGFR internalization than the sh-NC group (Fig. 6F, G). Additionally, co-localization analysis using immunofluorescence indicated that, following EGF stimulation, MAL2 knockdown facilitates the internalization of EGFR into the cytoplasm of ICC cells, where it co-localizes with the lysosomal degradation marker EEA1 (Fig. 6H). Conclusively, it appears that MAL2 can directly associate with EGFR, thus inhibiting EGFR endocytosis and subsequent degradation. This process facilitates the sustained activation of EGFR in response to EGF stimulation, which in turn, triggers the PI3K/AKT pathway and further bolsters lipid metabolism.

### Discovery of a small molecule inhibitor targeting the active site of MAL2

**Fig. 7:**
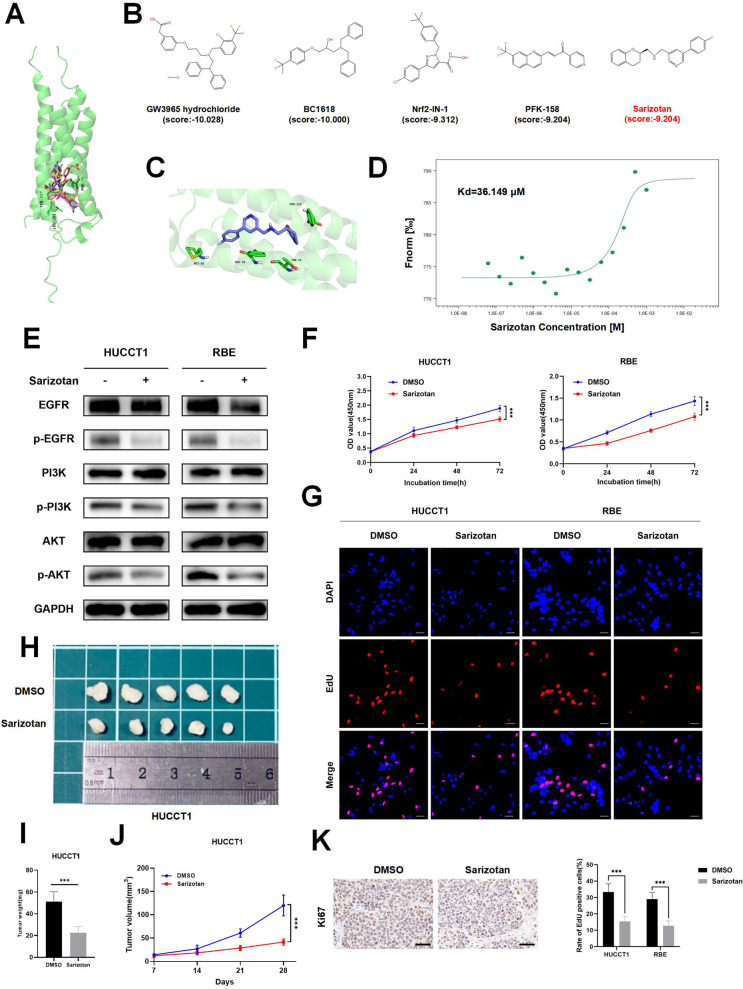
Identification of a minuscule molecular inhibitor aiming at MAL2’s active site. **A** The superimposed MAL2 protein (AlphaFold ID: AF-Q969L2-F1) alongside compounds chosen via computer-aided screening. **B** Conformational and docking advantages of sarizotan from the leading MAL2-compound scoring top-five interaction model. **C** A simplified depiction of sarizotan occupying the MAL2 protein’s active site. **D** MST analysis quantified the binding tenacity between sarizotan and MAL2. **E** The impact of sarizotan on the EGFR/PI3K/AKT signal pathway in HUCCT1 and RBE cells was explored using western blotting. **F** CCK-8 assay traced the growth trajectories of HUCCT1 and RBE cells under DMSO or sarizotan administration at 0, 24, 48, and 72 h intervals. **G** Cell propagation of ICC cells under DMSO or sarizotan treatment was scrutinized via EdU assay, scale bar, 50 μm. **H** Representative images of tumors from nude mice treated with DMSO or sarizotan. **I**, **J** Tumor growth curves and tumor weights of each group. **K** The expression level of Ki67 in nude mice-derived xenograft tumor was determined by IHC, scale bar, 50 μm. ***P < 0.001.

Given MAL2’s carcinogenic role in ICC, the discovery of inhibitors that target this protein has piqued significant interest. First, we posit that there could be key active sites susceptible to MAL2 inhibition. With the aid of Schrodinger Maestro 11.4, we predicted the potential active sites of these locales. The surface of MAL2, as per our technical speculations, possesses a pocket that might function as an active site housing critical residues, such as LEU101/PHE78/TYR105 (Fig. 7A). We then proceeded to bind more than 67,000 compounds to these forecasted locations. Based on the high-ranking MAL2-compound binding model (Fig. 7B), we selected and obtained the top-five compounds (namely, GW3965 hydrochloride, BC1618, Nrf2-IN-1, PFK-158, and Sarizotan). Within the sarizotan molecule, the pyridine ring is capable of forming a π–π interaction with the PHE78 amino acid residues of MAL2. Furthermore, sarizotan can form hydrophobic interactions with several MAL2 amino acid residues, including PHE70, VAL71, ALA75, PHE78, LEU81, MET85, and LEU101 (Fig. 7C). In the final stage, we utilized MST to measure the binding affinity (Kd) of the above-mentioned small molecule compounds to the MAL2 protein. The outcomes indicated that only sarizotan could assemble with MAL2, with a dissociation constant (Kd) indicative of a 36.149 μM binding affinity (Fig. 7D). According to the data from western blotting, sarizotan was observed to hinder EGFR/PI3K/AKT signal axis in HUCCT1 and RBE cells (Fig. 7E). As illustrated by the results of the CCK-8 and EdU assays, sarizotan reduced the proliferation rate of HUCCT1 and RBE cells (Fig. 7F, G). In vivo tests revealed that sarizotan successfully impeded tumor growth compared to the DMSO group (Fig. 7H–J). The immunohistochemical evidence also highlighted that sarizotan dramatically reduced the expression of Ki67 in tumor tissues (Fig. 7K). We further observed the toxicity of sarizotan by in vivo experiments in nude mice and found no significant toxicity in sarizotan-treated mice in terms of changes in body weight, AST and ALT levels in blood plasma, and any major organ system (Fig. S6A–C).

### MAL2 reduces sensitivity to cisplatin treatment in ICC both in vitro and in vivo

**Fig. 8:**
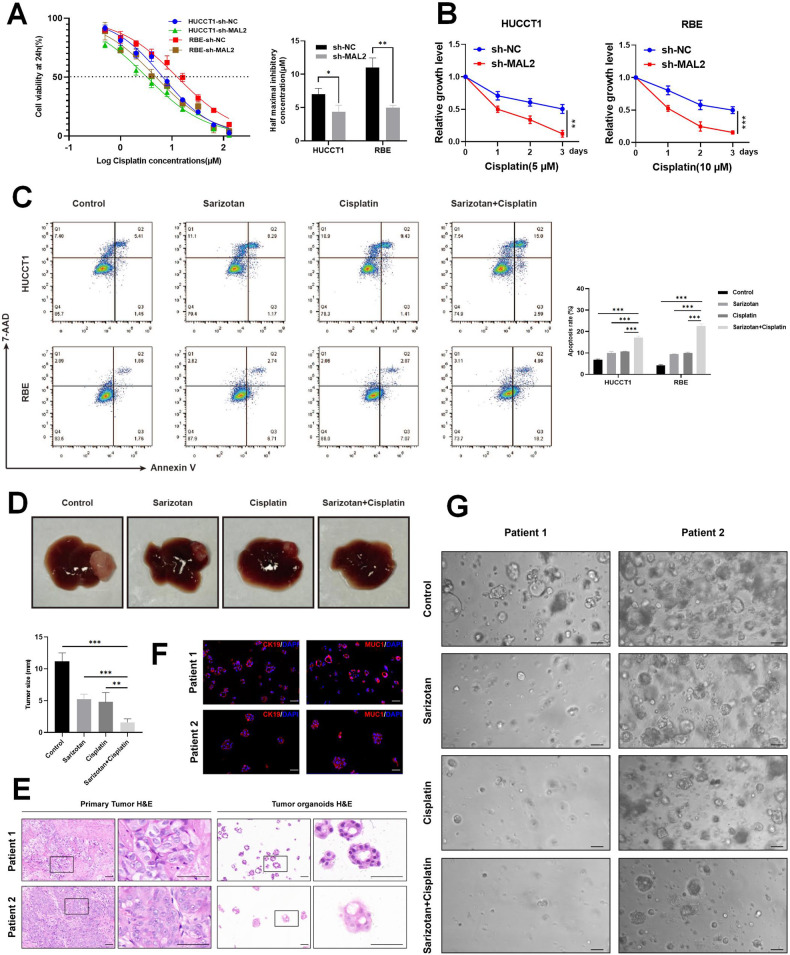
Sarizotan heightens the sensitivity of ICC to cisplatin. **A** Post 24 h of cisplatin treatment, the IC50 values for HUCCT1 and RBE cells transfected with sh-NC or sh-MAL2 were determined. **B** The survival of HUCCT1 and RBE cells, transfected with either sh-NC or sh-MAL2 was gauged via the CCK-8 assay following cisplatin administration (5 or 10 μM, respectively). **C** Apoptosis in HUCCT1 and RBE cells across various treatments was gauged with flow cytometry, employing the Annexin V/7AAD Apoptosis Detection Kit. **D** Macroscopic appearance of liver orthotopic transplanted tumor from different groups. **E** Depicted are representative images of primary tumors and their matching organoids stained with H&E, scale bar, 50 μm. **F** The immunofluorescence staining of CK19 and MUC1 (red) – the ICC markers, validated the retention of the primary tumor marker expression profile in the organoids, scale bar, 50 µm. **G** Canonical illustrations of two discrete ICC organoid lines post 72 h of varying treatments, scale bar, 200 μm. *P < 0.05; **P < 0.01; ***P < 0.001.

Given the propensity of patients to develop resistance to chemotherapy, and considering that cisplatin-based chemotherapy is the standard treatment regimen for advanced ICC, we were prompted to investigate if MAL2 has an influence on cisplatin sensitivity. We administered a range of cisplatin doses to HUCCT1 and RBE cells to establish the half-maximal inhibitory concentration (IC50) of cisplatin in ICC cell lines. In MAL2 knockdown cells, IC50 was reduced (Fig. 8A). In the CCK-8 experiments, we found that the inhibition of MAL2 decreased the survival and proliferation of cells under cisplatin exposure, whereas the overexpression of MAL2 resulted in the inverse effect (Fig. 8B and Fig. S7A). Furthermore, we observed that suppression of MAL2 enhanced cisplatin-induced cell death (Fig. S7B). Compared to the control group, sarizotan enhanced the apoptosis induced by cisplatin in HUCCT1 and RBE cells (Fig. 8C). We also established a mouse xenograft model to validate these findings in vivo. The administration of cisplatin has been demonstrated to be an efficient approach to halt tumor growth. The anti-proliferative effect of cisplatin was notably more evident in the tumors where MAL2 was knocked down (Fig. S7C–E). Immunohistochemical staining for MAL2, Ki67, and TUNEL indicated that the knockdown of MAL2 promoted cisplatin-induced apoptosis and reduced tumor proliferation (Fig. S7F). In an orthotopic transplantation model of ICC, the combination of sarizotan plus cisplatin produced much higher anticancer activity compared to either drug alone (Fig. 8D).

To further corroborate the influence of MAL2 on the susceptibility of ICC to cisplatin treatment, we developed an ICC organoid model. We employed hematoxylin and eosin (H&E) staining as well as immunofluorescence analysis to affirm the morphological and histological phenotypes of the ICC organoids, ensuring they mirrored primary tumors (Fig. 8E). As depicted in Fig. 8F, our immunofluorescence studies revealed that the ICC organoids expressed the ICC markers, CK19 and MUC1. The ICC organoid model yielded findings consistent with our prior experiments. Upon cisplatin treatment, the organoids with MAL2 knockdown exhibited a striking growth inhibition (Fig. S8A–C). Sarizotan also increased the growth inhibition induced by cisplatin in the ICC organoid model (Fig. 8G). Based on our findings, we draw the conclusion that the attenuation of MAL2 enhances the sensitivity of ICC to cisplatin.

## Discussion

ICC, a predominant form of primary liver cancer, is notably aggressive. Current medical interventions prove ineffectual for patients suffering from advanced local progression or metastatic manifestation of ICC. Even though research into chemotherapy and targeted therapies for ICC has seen progress, the survival rate for patients remains disappointingly low [1]. Therefore, the identification of molecular targets for the therapy of ICC is critical for enhancing therapeutic outcomes. The role of MAL2, known for its importance in tumor growth, in the pathogenesis of ICC has yet to be thoroughly explored. In this study, we present evidence that MAL2 is highly expressed in ICC and maintains EGFR’s membrane localization. This action subsequently activates the PI3K/AKT/SREBP-1 signaling pathway, leading to the upregulation of key fatty acid synthesis genes such as FASN and SCD, thereby promoting lipid accumulation. With the aid of virtual screening, we identified sarizotan, a small molecule inhibitor that targets MAL2. This compound has proven successful in inhibiting ICC growth and enhancing chemosensitivity, both in vitro and in vivo.

Research closely related to our investigation has indicated that MAL2 plays an essential role in the development of cancer and its presence often points to unfavorable outcomes for the patients. Zhang et al. [31] in their study, both in vitro and in vivo, found that MAL2 forms an interaction with IQGAP1 that causes an upregulation in ERK1/2 phosphorylation levels, thereby facilitating pancreatic cancer progression. Moreover, by modulating the epithelial–mesenchymal transition process, MAL2 might influence the migratory and invasive behaviors of cancer cells [32]. Additionally, the occurrence of MAL2-mediated endocytosis in the tumor environment has been found to inhibit the display of the MHC-I complex on breast cancer cells’ surface, thus reducing CD8+ T cell toxicity [8]. These previous findings underscore the role of MAL2 as a crucial molecular player in the development and progression of malignancy. However, an in-depth investigation into MAL2’s function and mechanism in ICC was missing until our study. Our research, underpinned by scRNA-seq analysis and immunohistochemical analysis of clinical specimens, revealed an overexpression of MAL2 in ICC, hinting at an unfavorable clinical prognosis. The suggestion of MAL2 as a prognostic factor in ICC cases underscores its importance as a biomarker in this malignancy.

Among the most evident metabolic alterations in cancer cells is the dysregulation of lipid metabolism. It is well documented that a variety of cancers manifest increased lipid intake, storage, and lipogenesis, which contribute to the rapid growth of the tumor [33]. As a part of ongoing efforts to uncover biomarkers that could aid in the detection, monitoring, and prognosis of cancer, lipid metabolism in CCA often comes under the scrutiny of metabolomic studies. Banales et al., for instance, leveraged serum metabolomics to pinpoint significant shifts in metabolites such as phosphatidylcholine, sphingolipids, and sterols present in the serum of CCA patients [34]. Furthermore, the lipid metabolites released by cancer cells could also remodel the tumor immune microenvironment especially their immunosuppressive cell components including tumor-associated macrophages, regulatory T cells and myeloid-derived suppressor cells [35]. In the context of our study, we found that when MAL2 was suppressed, there was a profound alteration in metabolites related to ICC lipid metabolism, with the most substantial impact on fatty acid synthesis. This observation, backed by metabolomic analysis, underscores the crucial role of lipid metabolic reprogramming in the pathogenesis of CCA, as corroborated by related studies. FASN is significantly associated with CCA progression as well as with poor patient prognosis, and purine metabolism is the most relevant metabolic change after FASN knockdown [13]. For instance, Zhang et al. [36] demonstrated that KDM5C could modulate the transcriptional alteration of FASN, thereby influencing fatty acid metabolism in ICC cells. circMBOAT2 is upregulated in ICC, and promotes cytoplasmic export of FASN mRNA through stabilization of PTBP1, which alters lipid metabolism and regulates redox homeostasis in ICC [37]. Hypoxia-induced SKA3 enhances fatty acid synthesis by regulating the PARP1/HIF-1α axis, which promotes CCA progression [38]. LncRNA HAGLROS is known to stimulate lipid metabolic reprogramming by changing the expression of key lipid metabolism genes in ICC cells [39]. The PI3K/AKT pathway plays a pivotal role in promoting fatty acid synthesis in cancer cells [40]. It has been found that SREBP-1, predominantly activated downstream of the PI3K/AKT axis in the oncogenic signaling pathway [41], regulates the expression of enzymes involved in lipid biosynthesis. As a membrane protein of the endoplasmic reticulum, SREBP-1 senses various metabolic signals and is activated by specific proteolytic processing [42]. After processing, a mature form of SREBP-1 translocates into the nucleus to stimulate transcription of its target genes, including those encoding key enzymes of the adipogenic pathway (e.g., ACLY, ACCAA, FASN, and SCD) [43]. eRO1A promotes migration and lipid metabolism in CCA cells through the AKT/mTOR/SREBP-1 signaling pathway [44]. EGFR, a tyrosine kinase receptor of the ErbB receptor family, activates PI3K/AKT in response to EGF stimulation [28]. Our investigation revealed that MAL2 associates with EGFR and aids in stabilizing EGFR membrane expression in ICC cells, as confirmed by Co-IP and immunofluorescence co-localization studies. Furthermore, we discovered that MAL2 inhibits EGFR endocytosis in response to EGF stimulation, which sequentially leads to the continuous activation of the PI3K/AKT/SREBP-1 signaling pathway, regulating the expression of critical genes involved in ICC fatty acid synthesis. Our study, therefore, provides a fresh perspective on the EGFR pathway.

With the development of modern molecular biology and the application of advanced technologies such as computer-aided drug design, structural biology, and combinatorial chemistry, small molecule-targeted anticancer drugs have entered a phase of rapid development. Specific small molecule inhibitors not only have the potential to simultaneously inhibit key oncogenic signaling pathways, but also inhibit the expression and/or activity of PD-L1 in specific cancers, making them attractive candidates for use in combination with existing immune checkpoint inhibitors and/or other targeted drugs [45]. The combination of CXCR2 antagonists with anti-PD1 in HCC suppressed tumor load and prolonged survival, and this combination therapy increased the activation of intratumoural XCR1 + dendritic cells and the number of CD8+ T cells, definitively strengthening anti-tumor immunity in HCC [46]. Another STAT3 small molecule inhibitor, LLL12, demonstrated significant anti-tumor effects in combination with cisplatin or paclitaxel, providing a viable insight into patients with ovarian cancer exhibiting sustained STAT3 signaling [47]. Small molecule inhibitors have become an important therapeutic class in tumor intervention due to the increasing understanding of molecular targets and their cellular functions [48]. Shiode et al. investigated hepatocyte transdifferentiation to promote ICC progression and found that the growth of a variety of human ICC cells in vitro and ICC xenografts in vivo was inhibited in vivo by the small molecule inhibitor NIK [49].

The elucidation of MAL2’s anti-tumor characteristics, as suggested by our investigations, might provide a fresh approach toward the treatment of ICC through the synthesis of drugs that can modulate MAL2 activity. Due to an increased understanding of molecular targets and their cellular functionality, small molecule inhibitors have become a prominent therapeutic class in oncological interventions. This preference for targeted therapeutic agents stems from their superior efficacy and safety compared to traditional chemotherapeutic agents [50, 51]. We proceeded with obtaining the predicted 3D structure of the human MAL2 protein (AlphaFold ID: AF-Q969L2-F1) from the AlphaFold database. Hydrogenation was performed on the protein using the Protein Preparation Wizard. This was succeeded by energy optimization applying the OPLS2005 force field and an RMSD of 0.30 Å. The optimized proteins underwent binding site prediction with the Sitemap module. Drawing upon these steps, virtual screening and in vivo external phenotyping were leveraged to screen and identify potential small molecule inhibitors. Among the screened molecules, sarizotan emerged as a novel compound demonstrating complete 5-HT1A agonist properties and significant affinity for the D3 and D4 receptors [52]. Preliminary evidence indicates that sarizotan has the ability to bind to the active site of MAL2, suggesting a potential anti-tumor role both in vitro and in vivo. Cisplatin, a widely utilized anti-tumor agent, is currently the first-line treatment for patients with advanced or metastatic ICC when used in combination therapies. However, one of the major impediments to successful treatment is the varying degrees of sensitivity and resistance to cisplatin among patients. In this context, our study unveils a novel finding – we observed that an overexpression of MAL2 attenuates the sensitivity of ICC to cisplatin and curbs apoptosis. When we employed sh-MAL2 and cisplatin treatment in organoid and CDX models of ICC, the results indicated that knocking down MAL2 heightens the responsiveness of ICC to cisplatin. Furthermore, our investigation revealed that when combined with cisplatin, sarizotan appears to amplify cisplatin-induced apoptosis in ICC cells, presenting a promising avenue for overcoming cisplatin resistance in clinical ICC.

## Conclusion

**Fig. 9:**
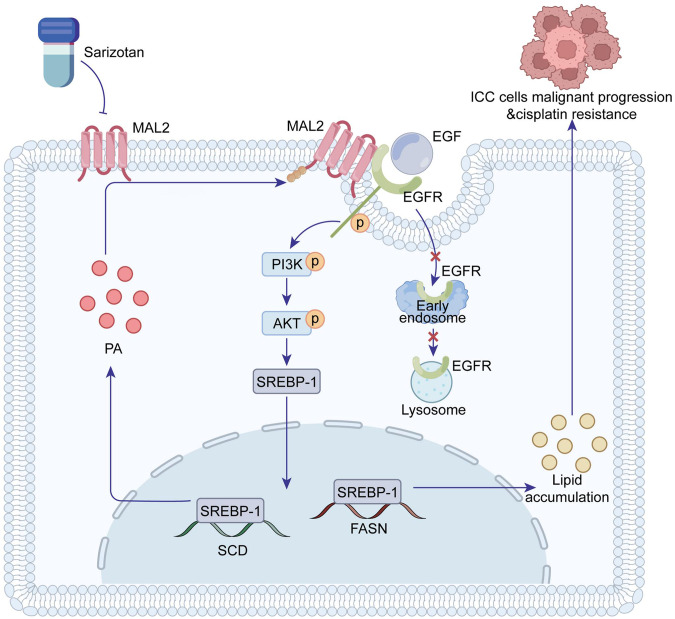
Charts the molecular modus operandi of MAL2 bolstering ICC advancement. When EGF is instigated, MAL2 sustains the membrane presence of EGFR, averting endocytosis, and persistently triggers the PI3K/AKT/SREBP-1 route. This sequence of events consequently accelerates lipid accretion in ICC and induces cisplatin insensitivity. Increased PA succeeds in preserving the membrane localization of MAL2. Sarizotan is identified as a diminutive molecular disruptor of MAL2, signifying its prospective utility in ICC therapy.

Our research unequivocally underscores the critical role of MAL2 as an oncogenic promoter in ICC. Upon exposure to EGF, MAL2 exhibits its ability to retain EGFR on the cell surface, thwarting the endocytosis process. This action consecutively triggers the PI3K/AKT/SREBP-1 signaling cascade, inciting an increase in lipid deposition within ICC cells. We also found that PA was capable of preserving the membrane-bound status of MAL2. Through meticulous screening, sarizotan emerged as a promising small molecule antagonist of MAL2. As such, our study suggests sarizotan as a viable therapeutic agent for ICC, with potential implications for augmenting cisplatin efficacy in future treatment strategies (Fig. 9).

### Supplementary information

Supplementary figure

Supplementary figure legends and tables

Original data file

## Data Availability

The authors declare that all data supporting the results in this study are available within the paper and its Supplementary Information. Source data for the figures in this study are available from the corresponding author upon reasonable request.
