# Supplementary figures and images for: MAL2 reprograms lipid metabolism in intrahepatic cholangiocarcinoma via EGFR/SREBP-1 pathway based on single-cell RNA sequencing
Source: Cell Death Dis. 2024 Jun 12;15(6):411. doi: 10.1038/s41419-024-06775-7 (PMC11169275; doi:10.1038/s41419-024-06775-7)

Figure S1

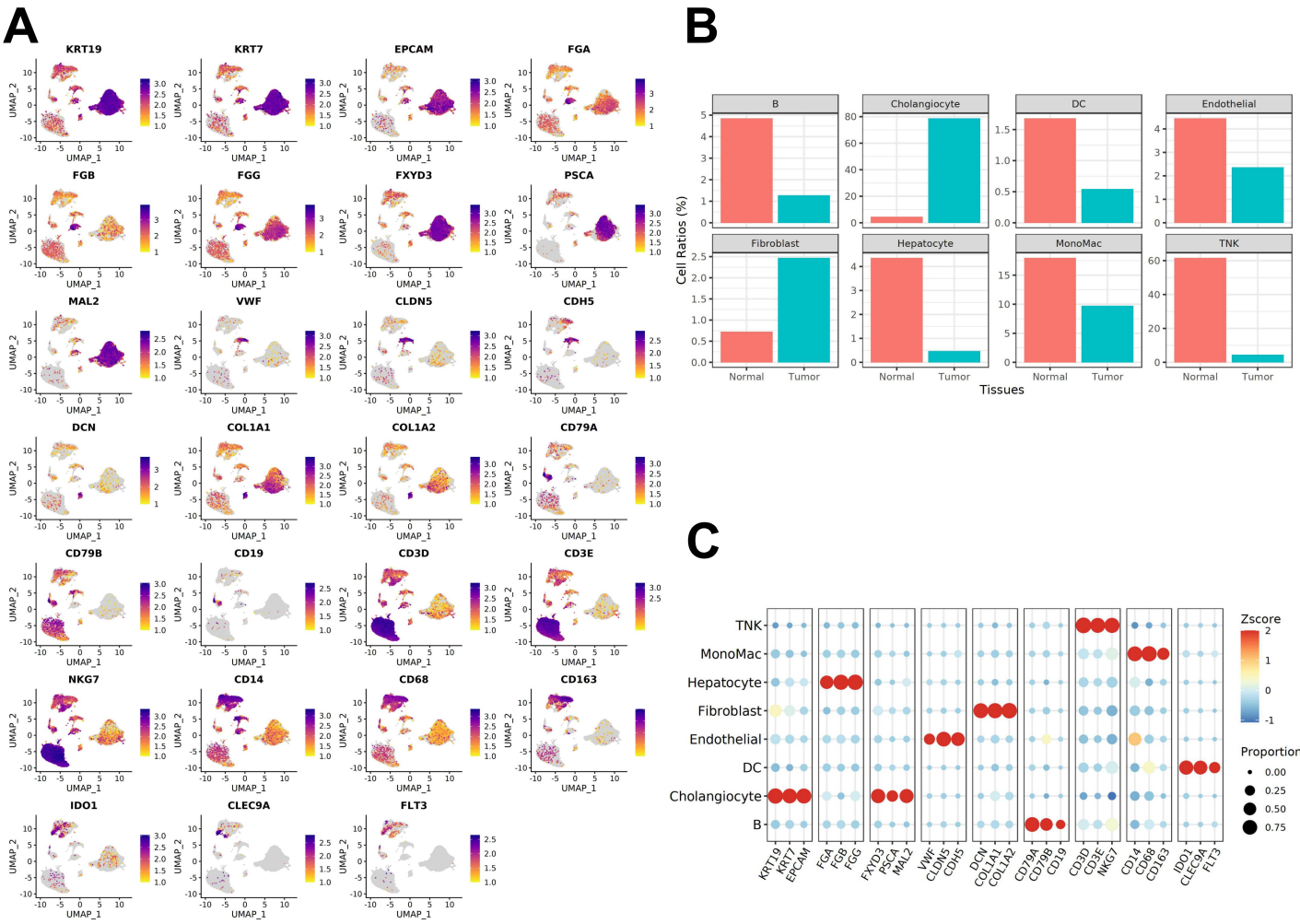

Figure S2

A

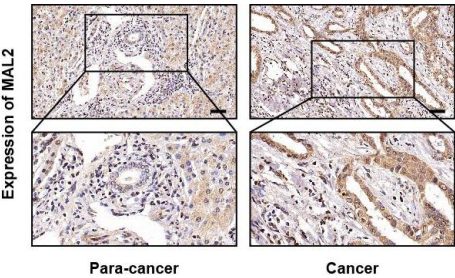

B

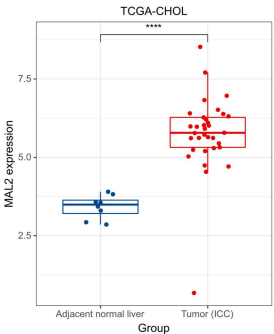

D

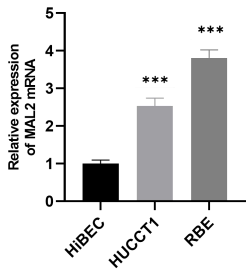

E

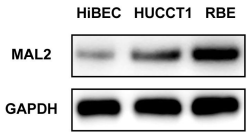

C

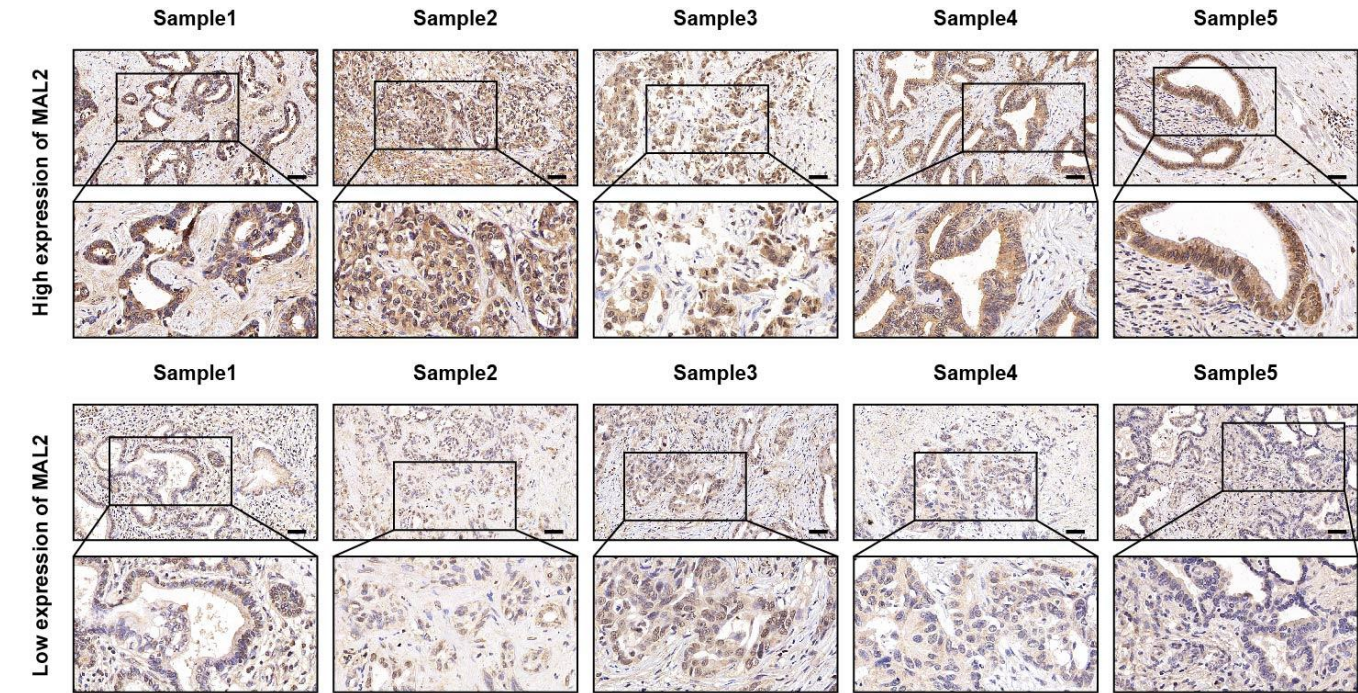

F

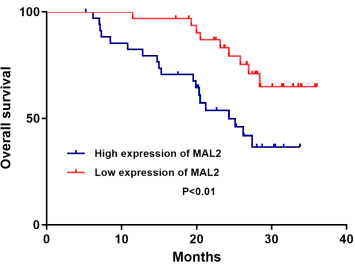

G

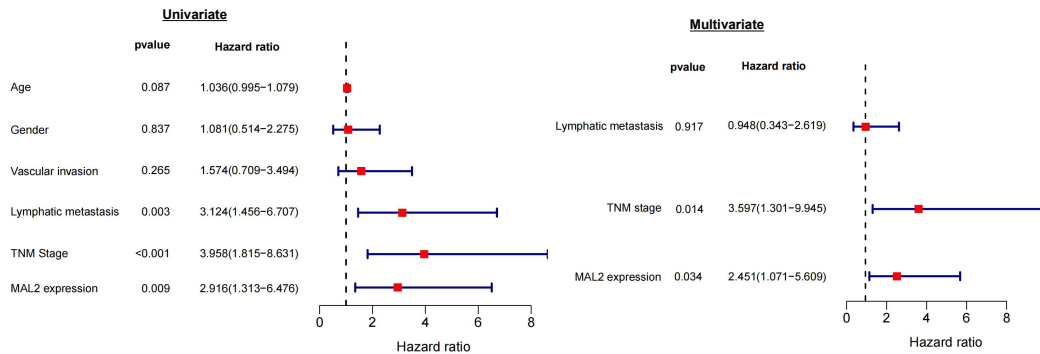

Figure S3

A

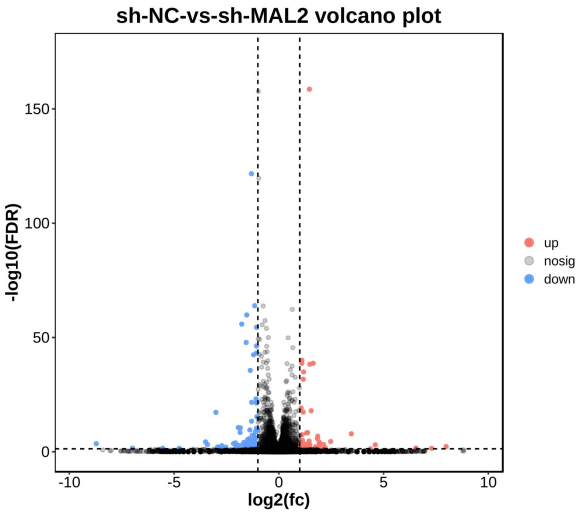

B

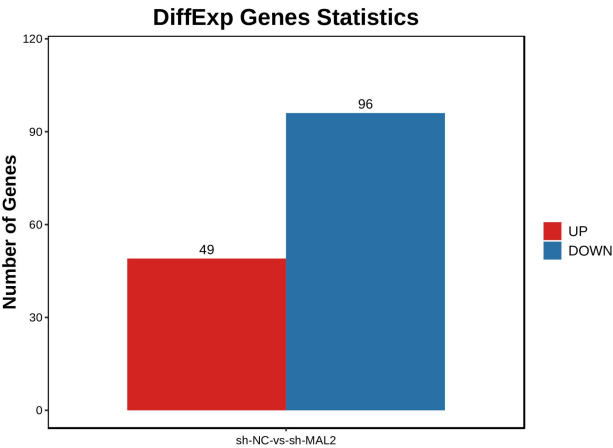

C

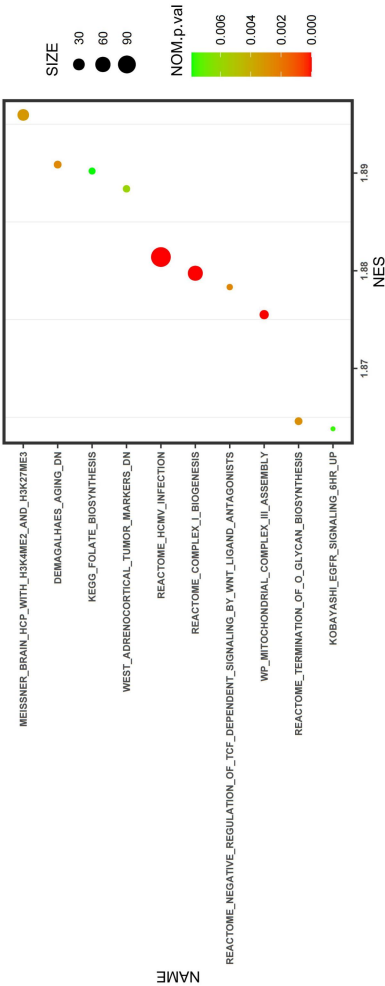

D

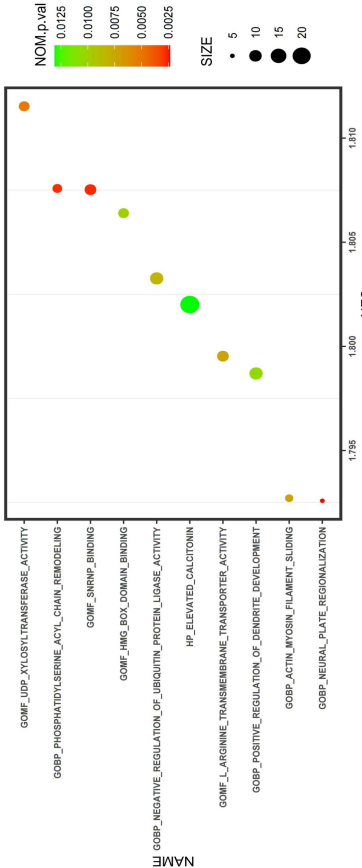

E

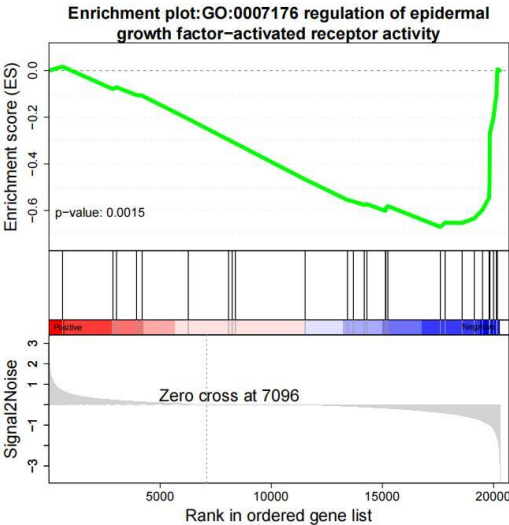

Figure S4

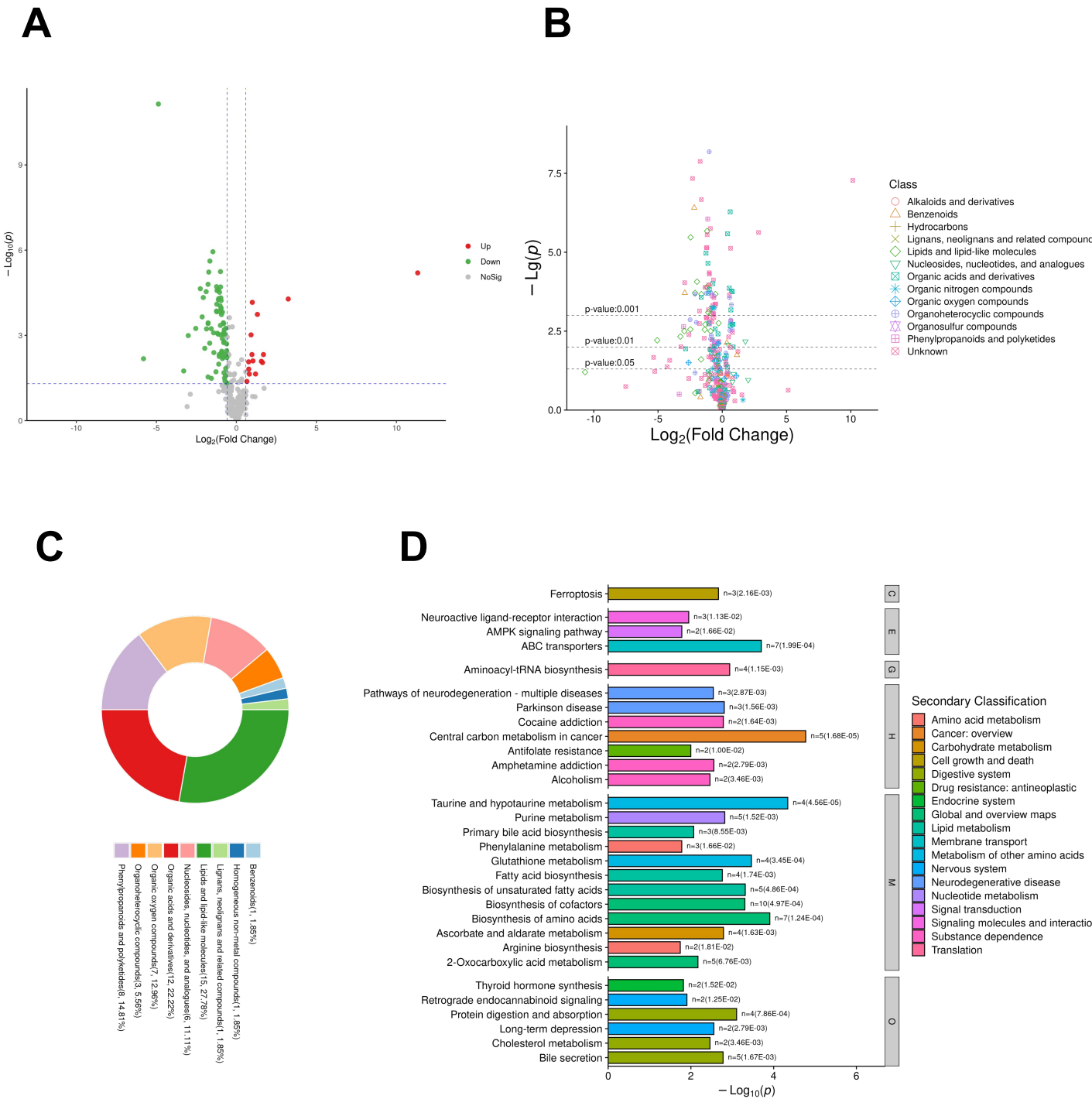

Figure S5

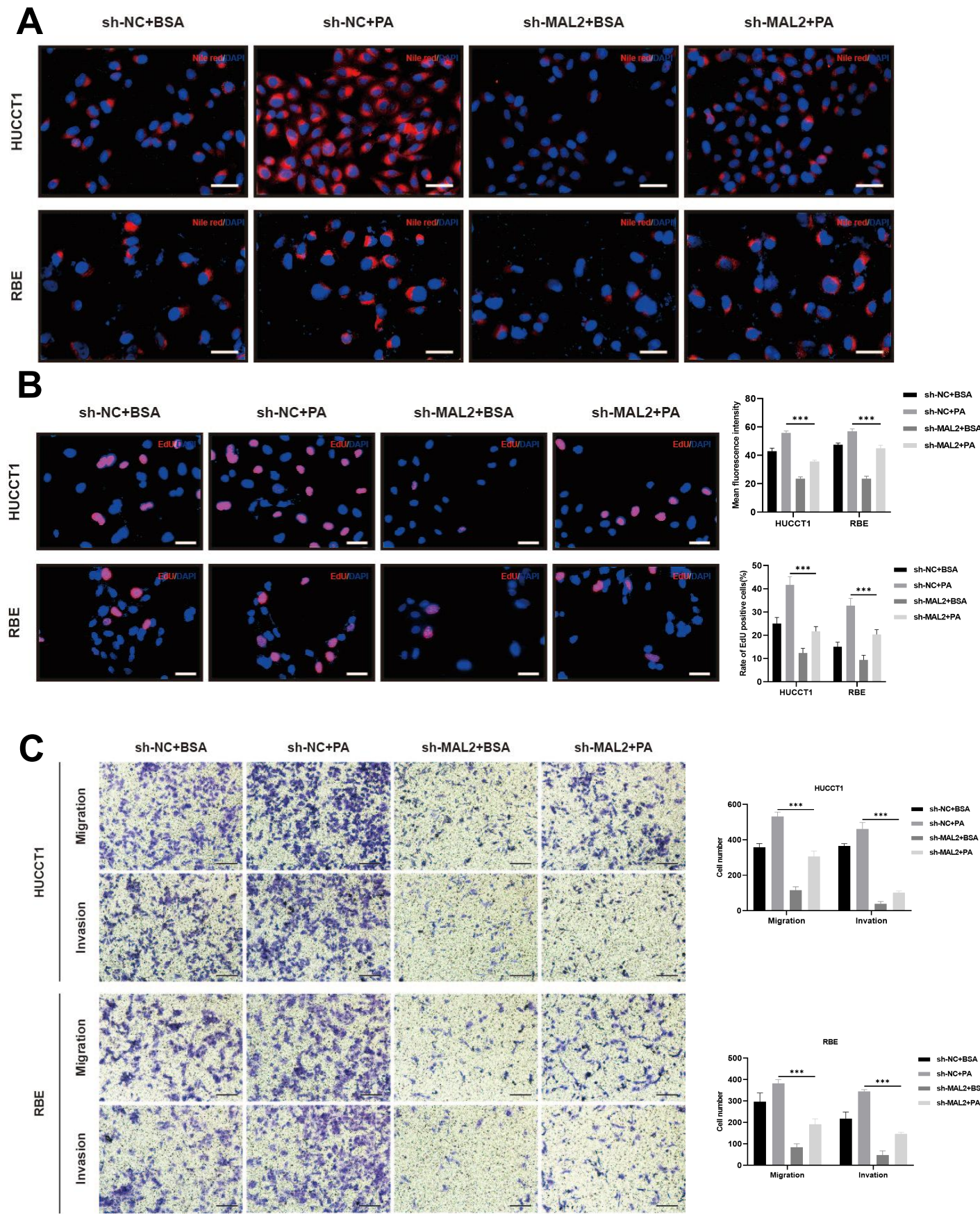

Figure S6

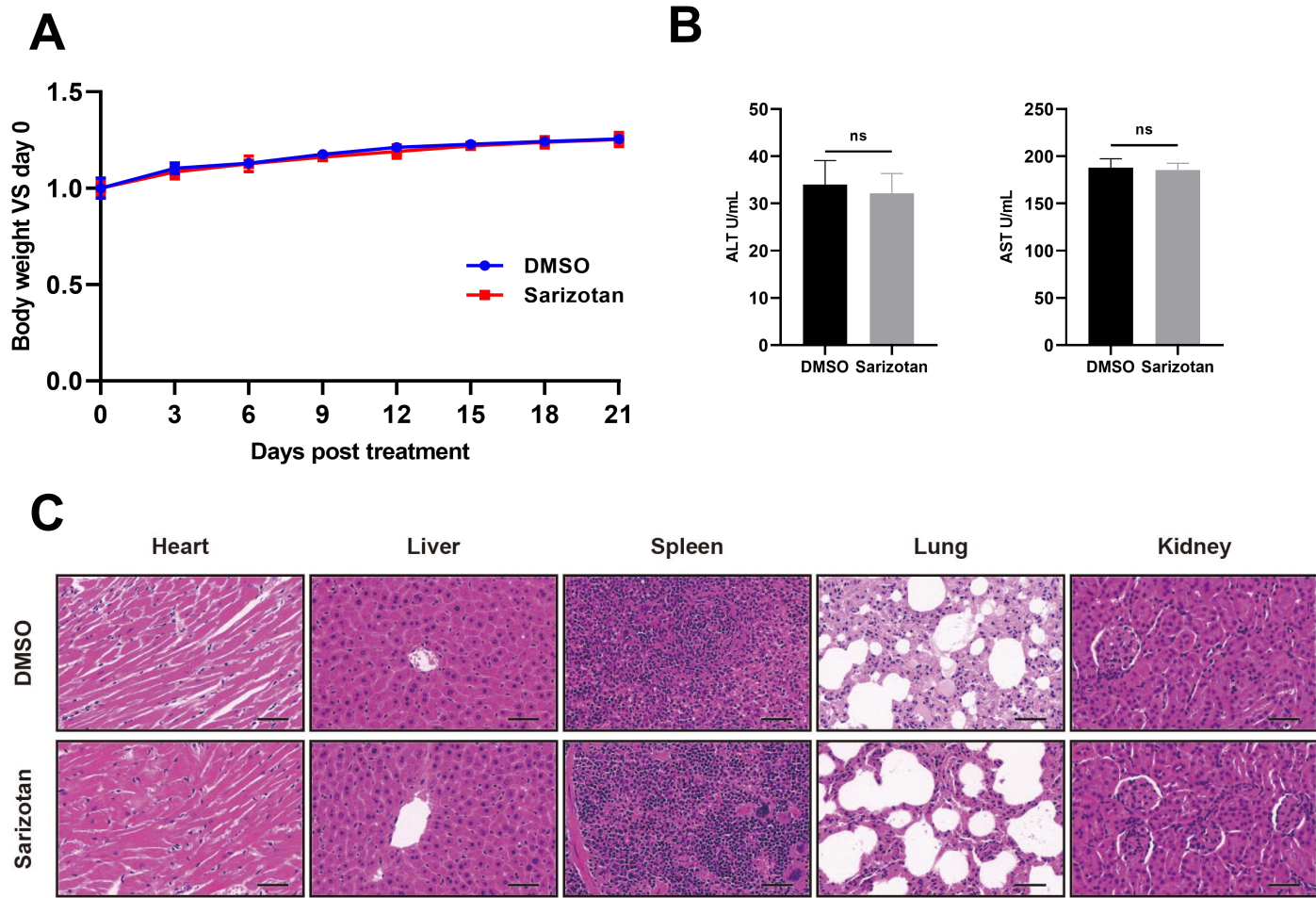

Figure S7

A

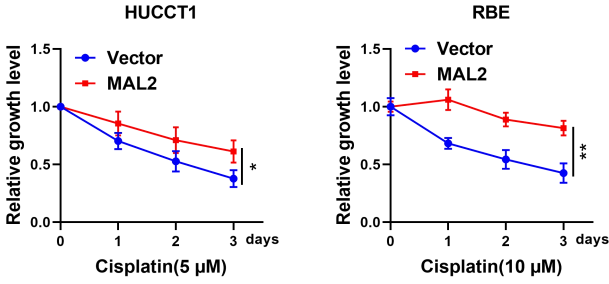

B

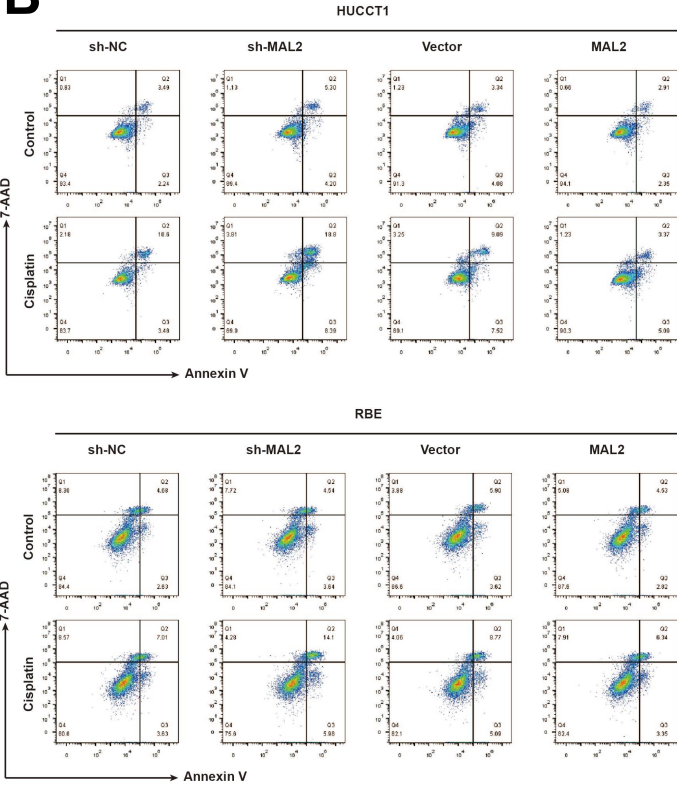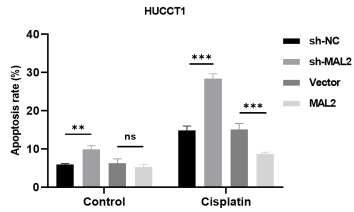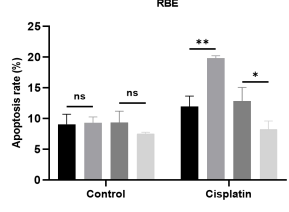

C

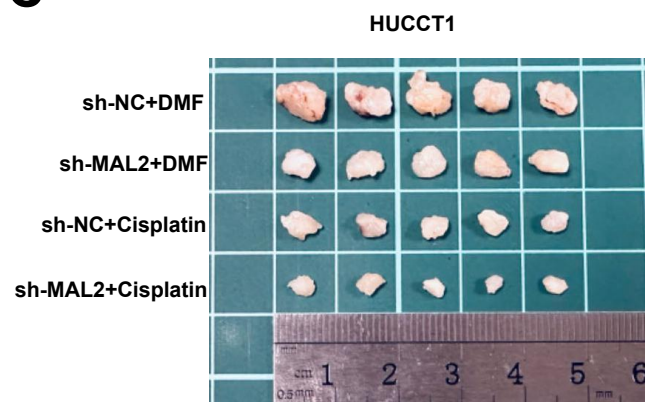

D

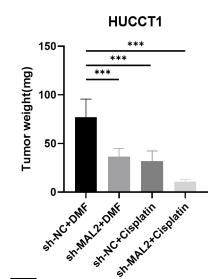

E

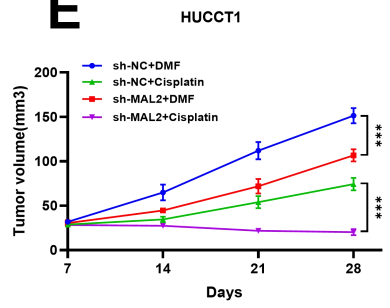

F

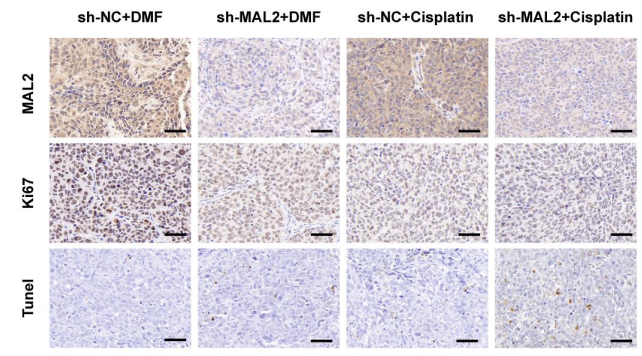

Figure S8

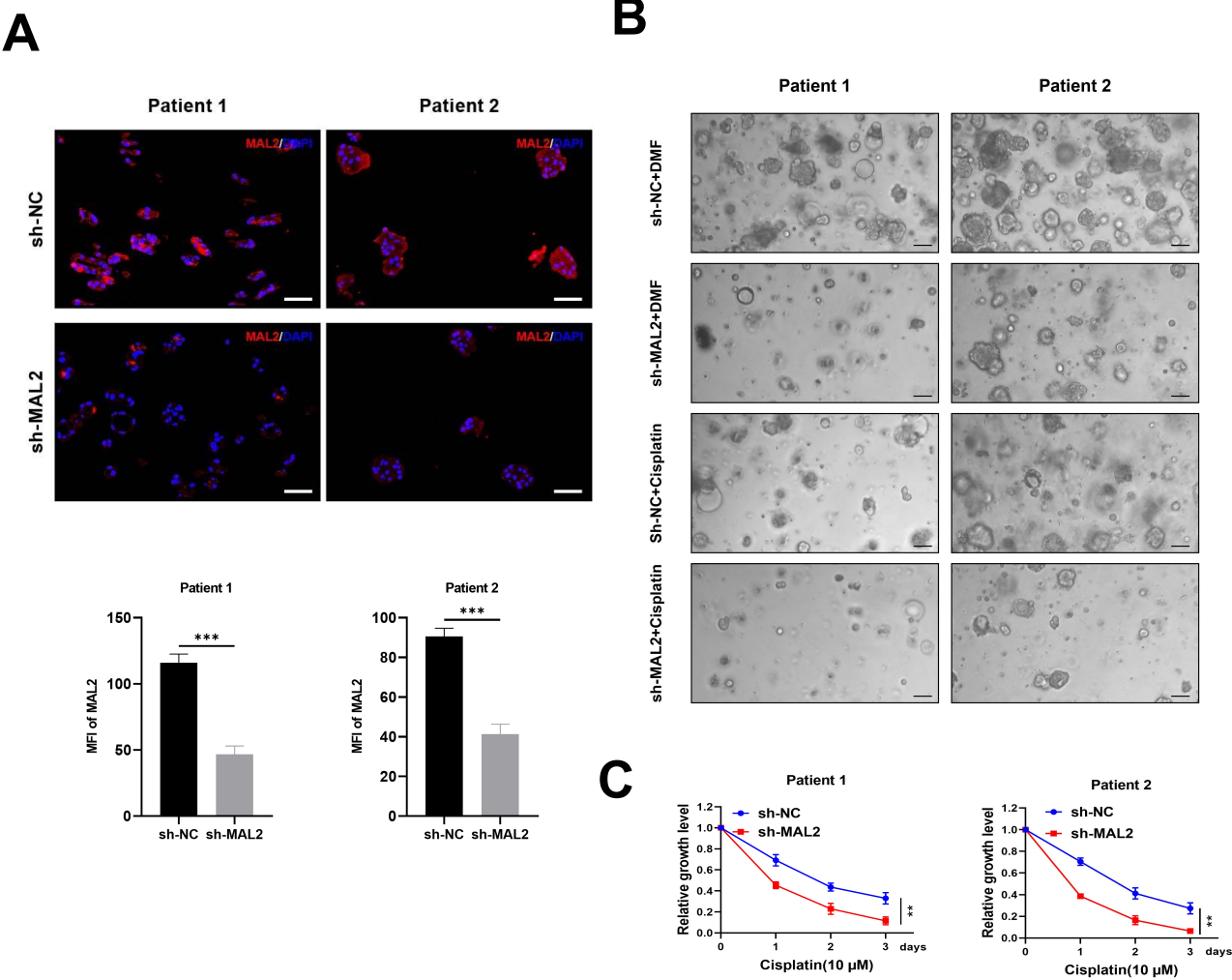

Supplement: Supplementary file 1 — Supplementary figure [file 41419_2024_6775_MOESM1_ESM.pdf]
