# Supplementary material for: MAL2 reprograms lipid metabolism in intrahepatic cholangiocarcinoma via EGFR/SREBP-1 pathway based on single-cell RNA sequencing
Source: Cell Death Dis. 2024 Jun 12;15(6):411. doi: 10.1038/s41419-024-06775-7 (PMC11169275; doi:10.1038/s41419-024-06775-7)
Supplement: Supplementary file 2 — Supplementary figure legends and tables [file 41419_2024_6775_MOESM2_ESM.docx]

**Supplementary figure legends:**

**Figure S1:**

(A) UMAP displaying different marker expression in different cell clusters.

(B) The bar chart illustrating the variations in expression of the different cell populations present in the different tissues.

(C) Dot plot showing different cell populations and the markers that distinguish them.

**Figure S2:**

1. Immunohistochemistry demonstrating MAL2 expression in cancer tissue and paracancer tissues, scale bar, 20μm.
2. TCGA showing MAL2 expression in normal and cancer tissues.
3. Representative immunohistochemical images showing high and low expression of MAL2 in ICC tissues, scale bar, 20μm.
4. The relative mRNA expression of MAL2 in HiBEC or HUCCT1 and RBE cells was scrutinized via qRT-PCR.
5. Quantification of MAL2 protein expression in HiBEC or HUCCT1 and RBE cells using western blotting technique.

(F) Relationship between MAL2 expression and overall survival in ICC patients.

(G) Univariate and multivariate Cox proportional risk analysis to assess the impact of MAL2 on overall survival in patients with ICC. *P < 0.05; **P < 0.01; ***P < 0.001.

**Figure S3:**

(A) Volcanic maps showing changes in gene expression after the knockdown of MAL2 in ICC cells.

(B) Statistical map of up-regulated or down-regulated differential genes.

(C-D) The enrichment of KEGG pathways and Gene Ontology (GO) terms among differentially expressed genes.

(E) The GSEA revealed a significant enrichment of regulation of epidermal growth factor-activated receptor activity.

**Figure S4:**

(A-B) Volcano map showing differential metabolites and classifying metabolites by HMDB.

(C) Proportion of different types of differential metabolites.

(D) KEGG pathway analysis indicated the main concentrated pathways of these differential metabolites.

**Figure S5:**

1. The baseline lipid droplet (LD) content in sh-NC or sh-MAL2 HUCCT1 and RBE cells post PA treatment (100 μM, 24 h) was evaluated using Nile red staining, scale bar, 50μm. This was followed by quantifying the mean fluorescence intensity of Nile red staining for each cell line.
2. Cell proliferation in ICC cells transfected with sh-NC or sh-MAL2 post PA treatment (100 μM, 24 h) was evaluated using an EdU assay; the scale bar represents 50μm.
3. Invasion potential of HUCCT1 and RBE cells transfected with sh-NC or sh-MAL2 post PA treatment (100 μM, 24 h) was investigated using a Transwell invasion assay; the scale bar stands for 200μm. *P < 0.05; **P < 0.01; ***P < 0.001.

**Figure S6:**

1. The body weight was measured and recorded every 3 days.
2. AST and ALT levels in blood plasma of each group.
3. H&E staining of histological sections from major organs, including heart, liver, spleen, lung and kidney, scale bar, 50μm. *P < 0.05; **P < 0.01; ***P < 0.001.

**Figure S7:**

1. The survival of HUCCT1 and RBE cells, transfected with either control or MAL2 vector, was gauged via the CCK-8 assay following cisplatin administration (5 or 10 μM respectively).

(B) After a 24-hour exposure to DMF (control) or cisplatin (5 or 10 μM, respectively), cell apoptosis in HUCCT1 and RBE cells was assessed through flow cytometry utilizing an Annexin V/7AAD Apoptosis Detection Kit.

(C) Illustrative images of tumors procured from nude mice after assorted treatments.

(D-E) Progression of tumor growth and respective tumor weights for each group.

(F) Immunohistochemistry was employed to ascertain the expression levels of MAL2, Ki67, and TUNEL in xenograft tumors derived from nude mice, scale bar, 50μm. * P < 0.05; **P < 0.01; ***P < 0.001.

**Figure S8:**

1. The levels of MAL2 within ICC organoids were ascertained using immunofluorescence, scale bar, 50µm.
2. Illustrative images of a pair of distinct ICC organoid lines post a 72-hour treatment with either DMF or cisplatin (10 μM), scale bar, 200μm.
3. The survival rate of sh-NC or sh-MAL2 transfected ICC organoids that were treated with cisplatin (10 μM) was evaluated based on the CCK-8 assay. * P < 0.05; **P < 0.01; ***P < 0.001.

**Table S1**. **Primer sequences, shRNAs used in this study**

| **Primer/shRNA** | | **Sequence (5’-3’)** | |
| --- | --- | --- | --- |
| human-MAL2 | Forward primer | | GTCCGTGACAGCGTTTTTCTT |
|  | Reverse primer | | AATTGAGGCTGCTACGTTTATGT |
| human-ACLY | Forward primer | | ATCGGTTCAAGTATGCTCGGG |
|  | Reverse primer | | GACCAAGTTTTCCACGACGTT |
| human-ACACA | Forward primer | | ATGTCTGGCTTGCACCTAGTA |
|  | Reverse primer | | CCCCAAAGCGAGTAACAAATTCT |
| human-SCD | Forward primer | | GCCCCTCTACTTGGAAGACGA |
|  | Reverse primer | | AAGTGATCCCATACAGGGCTC |
|  | Forward primer | | GGACCTGACCTGCCGTCTAG |
| human-GAPDH | Reverse primer | | GTAGCCCAGGATGCCCTTGA |
| human-FASN | Forward primer | | AAGGACCTGTCTAGGTTTGATGC |
|  | Reverse primer | | TGGCTTCATAGGTGACTTCCA |
| human-sh1-MAL2 | ccggcgACAGCTTGTTATGGTTGCActcgagTGCAACCATAACAAGCTGTcgtttttg | | |
| human-sh2-MAL2 | ccggccTGCATGATTTGCATTGCAActcgagTTGCAATGCAAATCATGCAggtttttg | | |
| human-sh3-MAL2 | ccggctAACTGGAACTTCCTGGATTctcgagAATCCAGGAAGTTCCAGTTagtttttg | | |

**Table S2**. **Antibodies in this study**

| **Name** | **Supplier** | **Cat no.** |
| --- | --- | --- |
| MAL2 | BIOSS | bs-7175R |
| GAPDH | Cell Signaling Technology | #5174 |
| PI3 Kinase | Cell Signaling Technology | #4257 |
| Phospho-PI3 Kinase | Cell Signaling Technology | #17366 |
| AKT | Cell Signaling Technology | #4691 |
| p-AKT | Cell Signaling Technology | #13038 |
| Flag | Abcam | ab205606 |
| HA | Abcam | ab9110 |
| EGFR | Abcam | ab52894 |
| p-EGFR | Abcam | ab40815 |
| SREBP1 | BIOSS | bs-1402R |
| Na,K-ATPase | ABclonal | A24883 |
| EGFR | BIOSS | bsm-10695M |
| HRP-linked anti-rabbit IgG | Cell Signaling Technology | #7074 |
| HRP-linked anti-mouse IgG | Cell Signaling Technology | #7076 |
| Alexa Fluor^TM^ 488 | Abcam | Ab150077 |
| Alexa Fluor^TM^ 594 | Abcam | Ab150116 |
| EGFR/PE | BIOSS | bs-0165R-PE |

**Table S3. Clinical characteristics of 67 patients with ICC**

| **Characteristics** | **No. of patients** | **MAL2 expression** | | **P value** |
| --- | --- | --- | --- | --- |
|  | **N= 67** | **Low (N= 32)** | **High (N= 35)** |  |
| **Age(year)** |  |  |  | 0.4996 |
| ≥60 | 36 | 15 | 21 |  |
| <60 | 31 | 17 | 14 |  |
| **Gender** |  |  |  | 0.1889 |
| Female | 32 | 18 | 14 |  |
| Male | 35 | 14 | 21 |  |
| **Vascular invasion** |  |  |  | 0.5667 |
| Yes | 19 | 8 | 11 |  |
| No | 48 | 24 | 24 |  |
| **Lymphatic metastasis** |  |  |  | 0.0480* |
| Positive | 18 | 5 | 13 |  |
| Negative | 49 | 27 | 22 |  |
| **TNM Stage** |  |  |  | 0.0303* |
| I-II | 39 | 23 | 16 |  |
| III | 28 | 9 | 19 |  |

**Note: *P<0.05**
