# Supplementary material for: MAL2 reprograms lipid metabolism in intrahepatic cholangiocarcinoma via EGFR/SREBP-1 pathway based on single-cell RNA sequencing
Source: Cell Death Dis. 2024 Jun 12;15(6):411. doi: 10.1038/s41419-024-06775-7 (PMC11169275; doi:10.1038/s41419-024-06775-7)

Full and uncropped western blot for Figure 2B-C

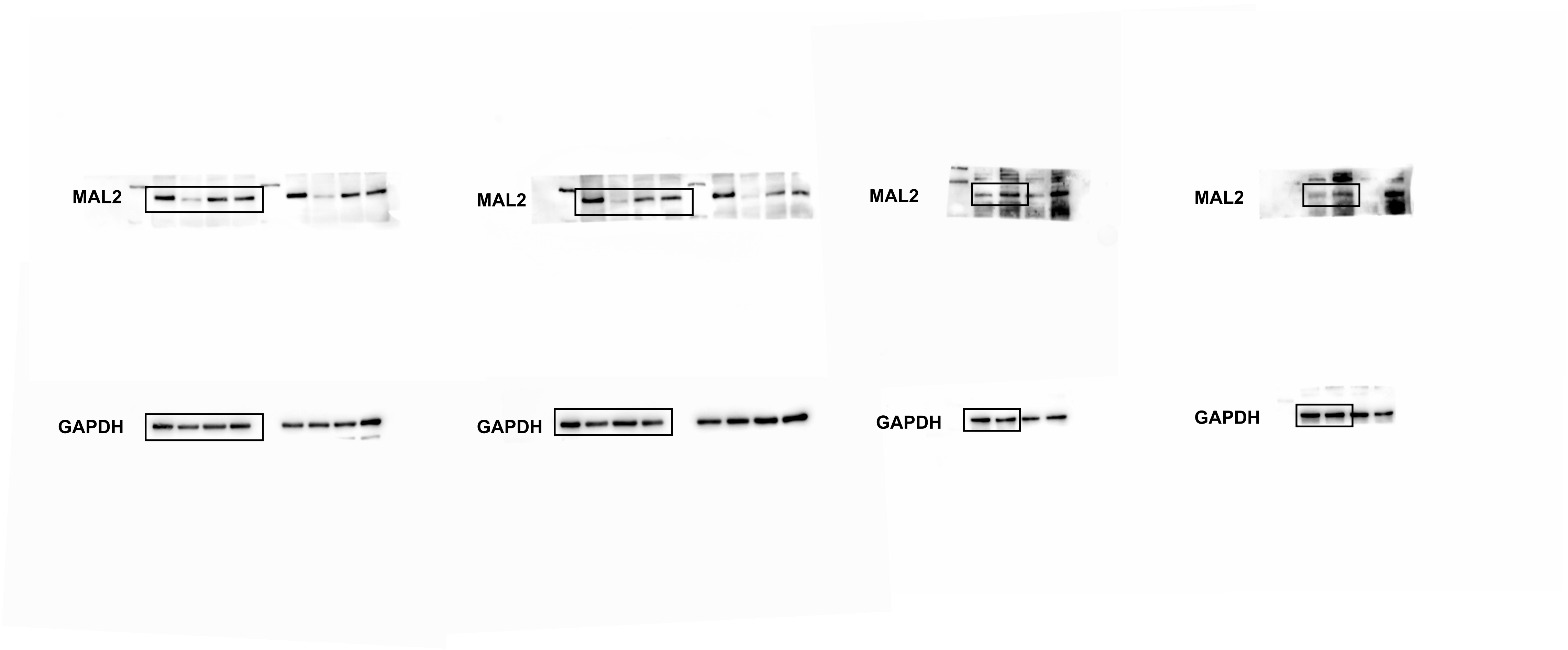

Full and uncropped western blot for Figure 4C

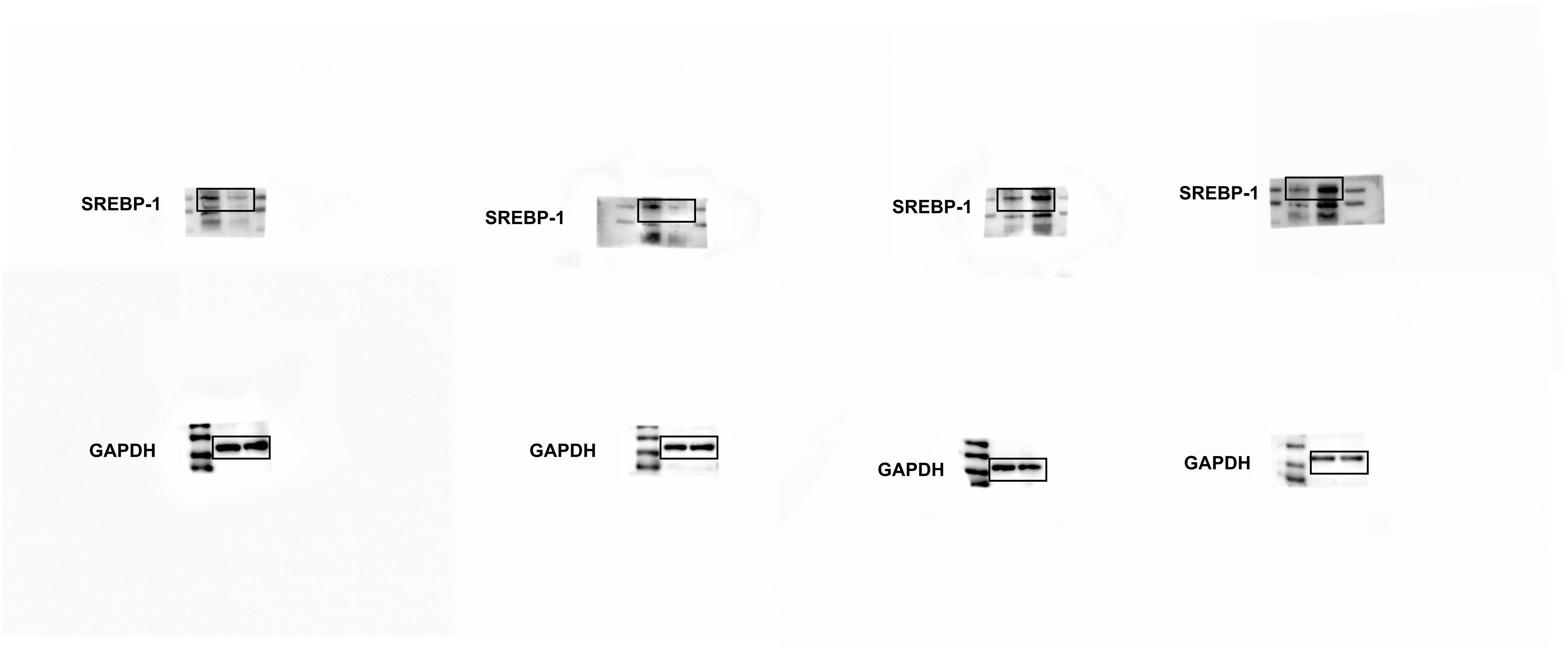

Full and uncropped western blot for Figure 4F

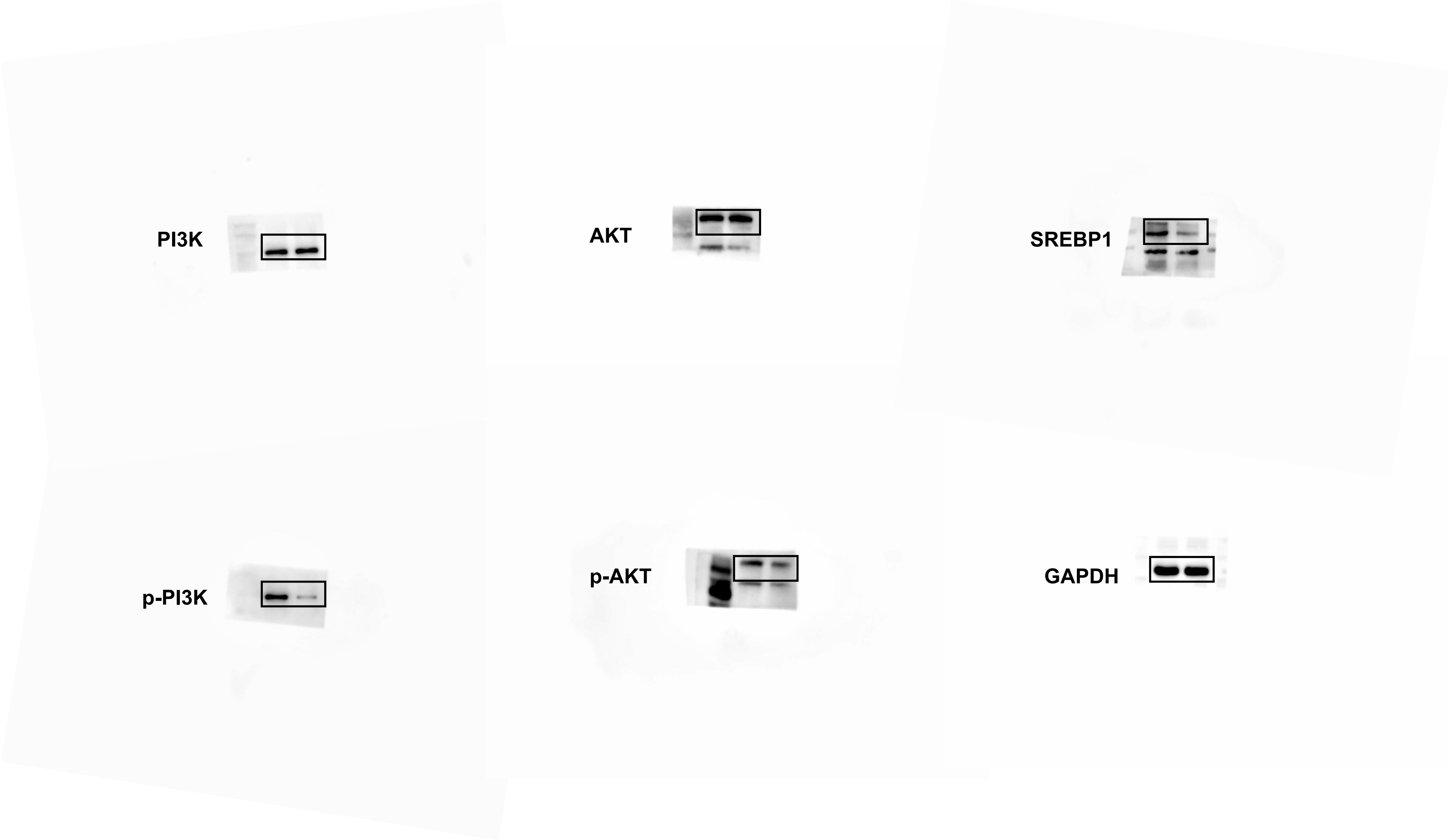

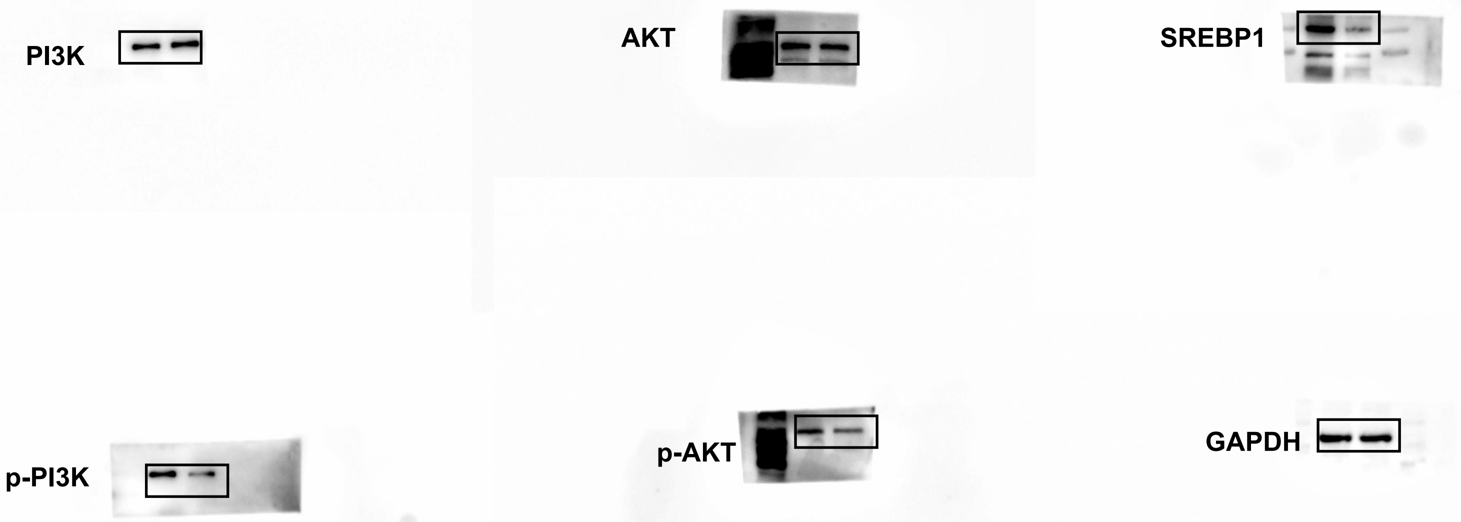
PI3K
AKT
SREBP1
p-PI3K
p-AKT
GAPDH

Full and uncropped western blot for Figure 4H-I

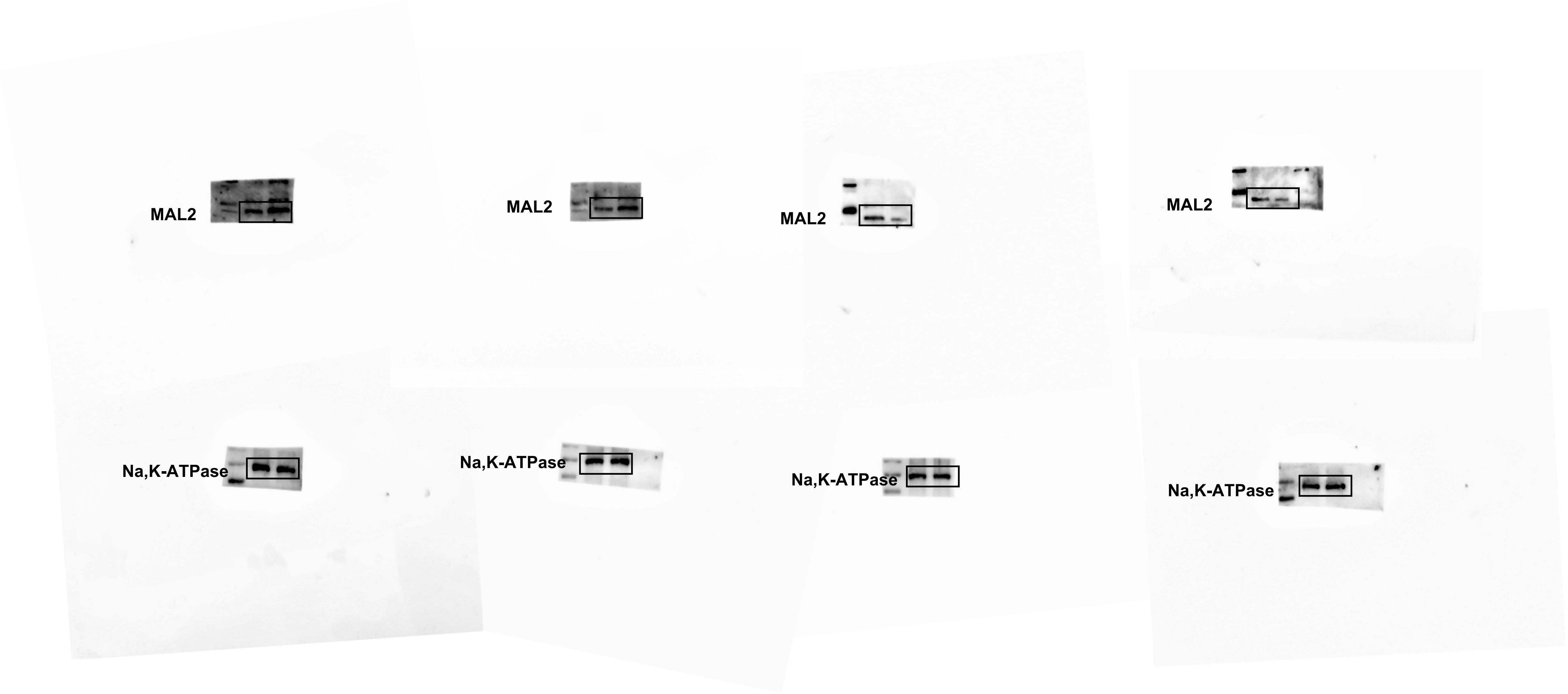

Full and uncropped western blot for Figure 5D

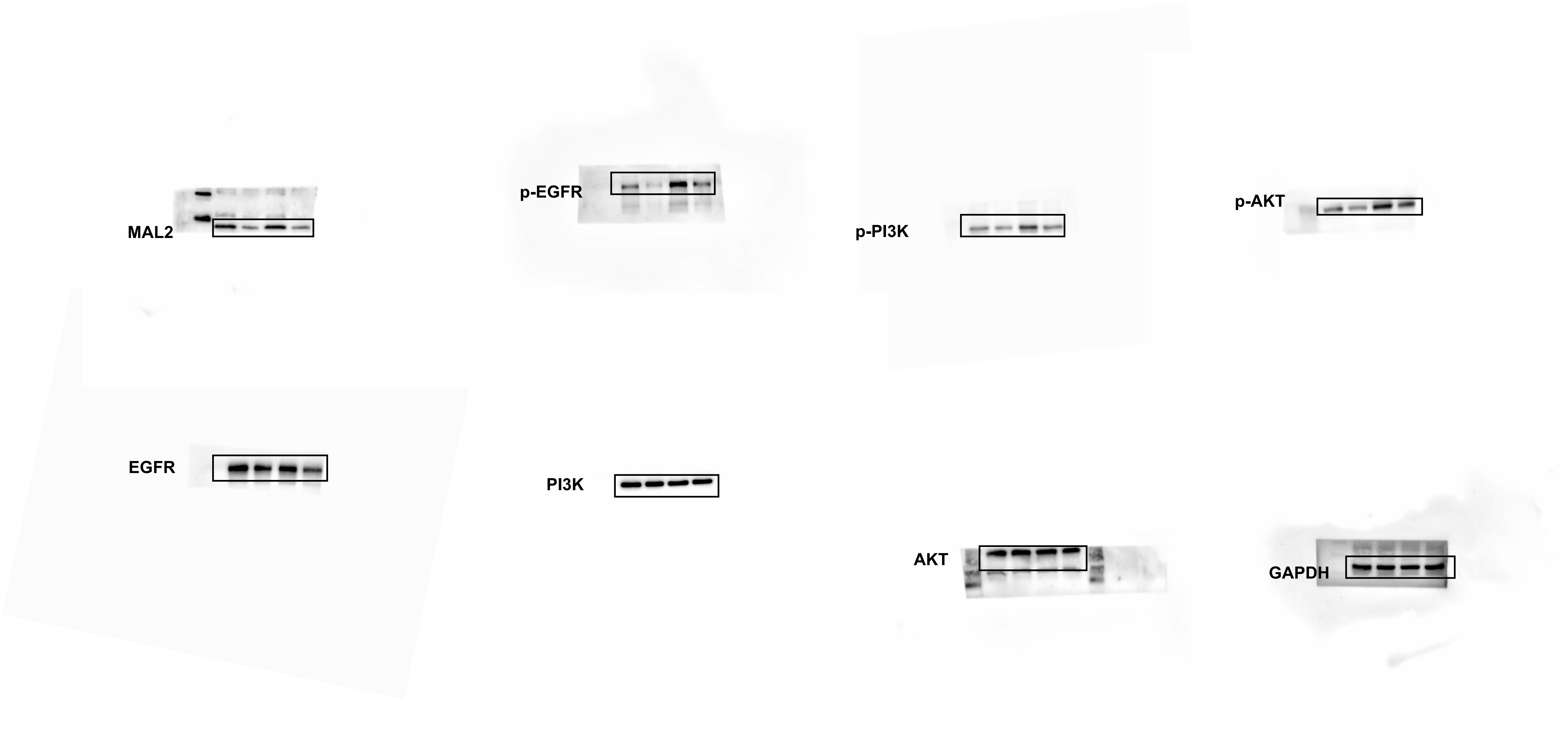

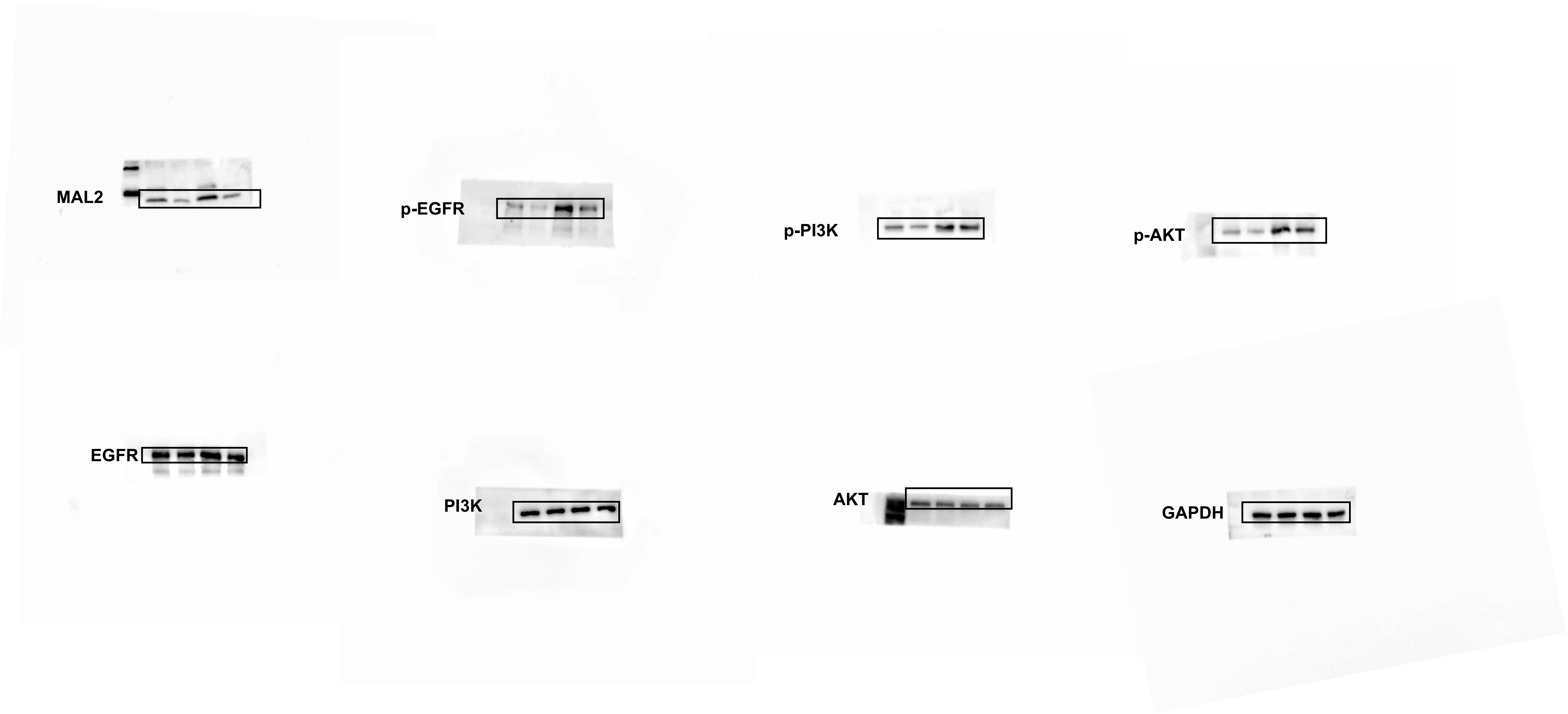
MAL2
p-EGFR
p-PI3K
p-AKT
EGFR
PI3K
AKT
GAPDH

Full and uncropped western blot for Figure 5E

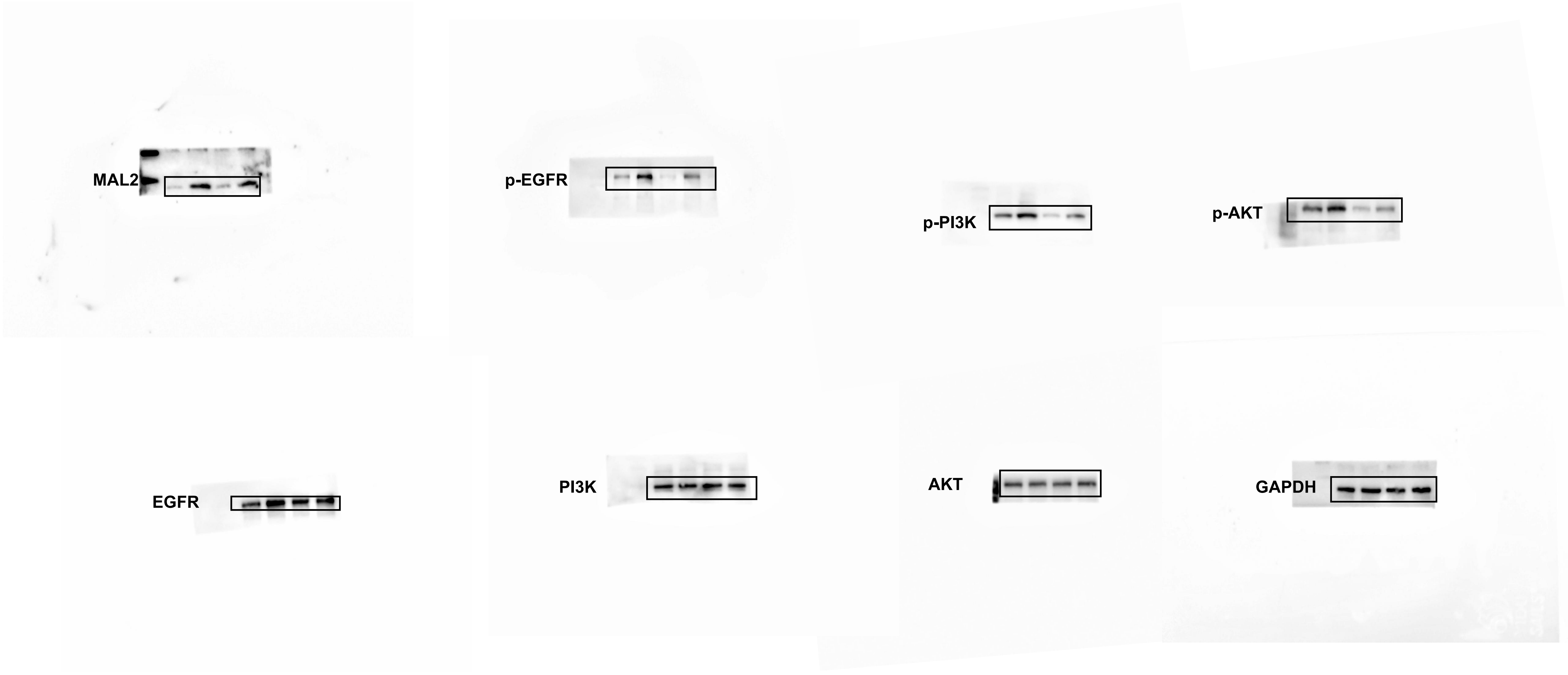

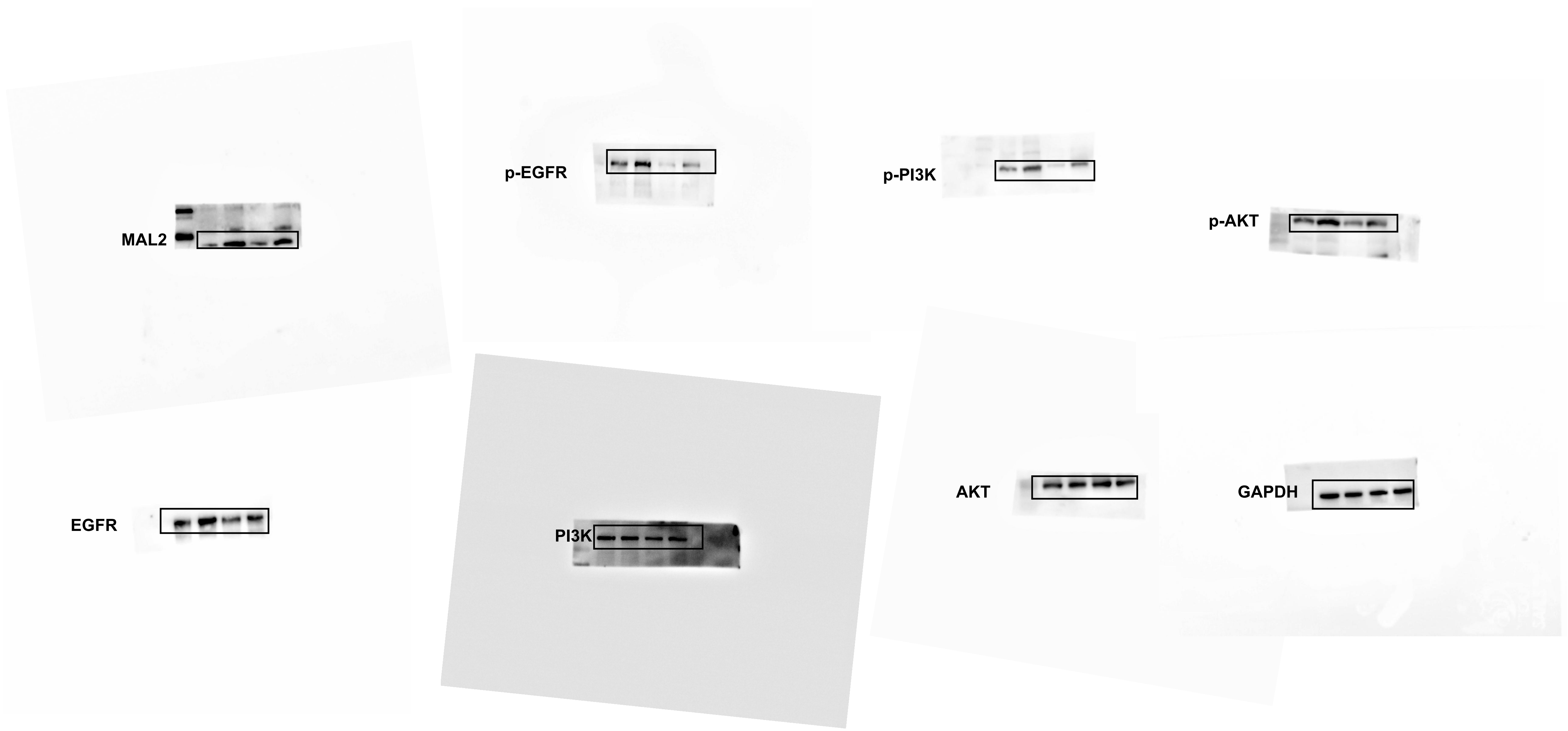
MAL2
p-EGFR
p-PI3K
p-AKT
EGFR
PI3K
AKT
GAPDH

Full and uncropped western blot for Figure 6B

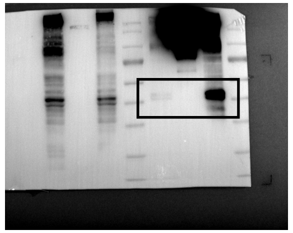

HA

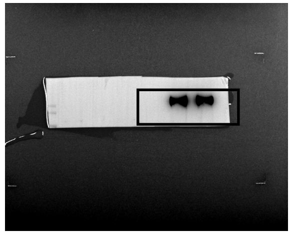

FLAG

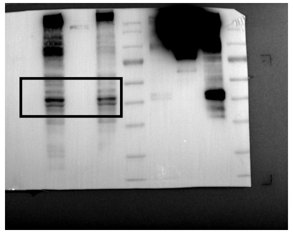

HA

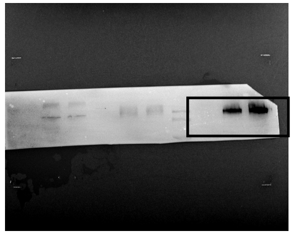

FLAG

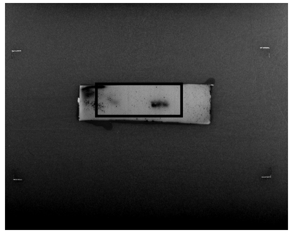

FLAG

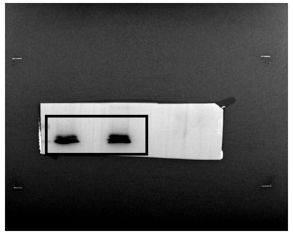

HA

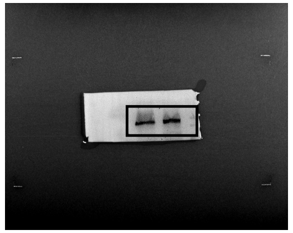

FLAG

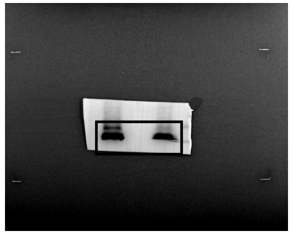

HA

Full and uncropped western blot for Figure 6D

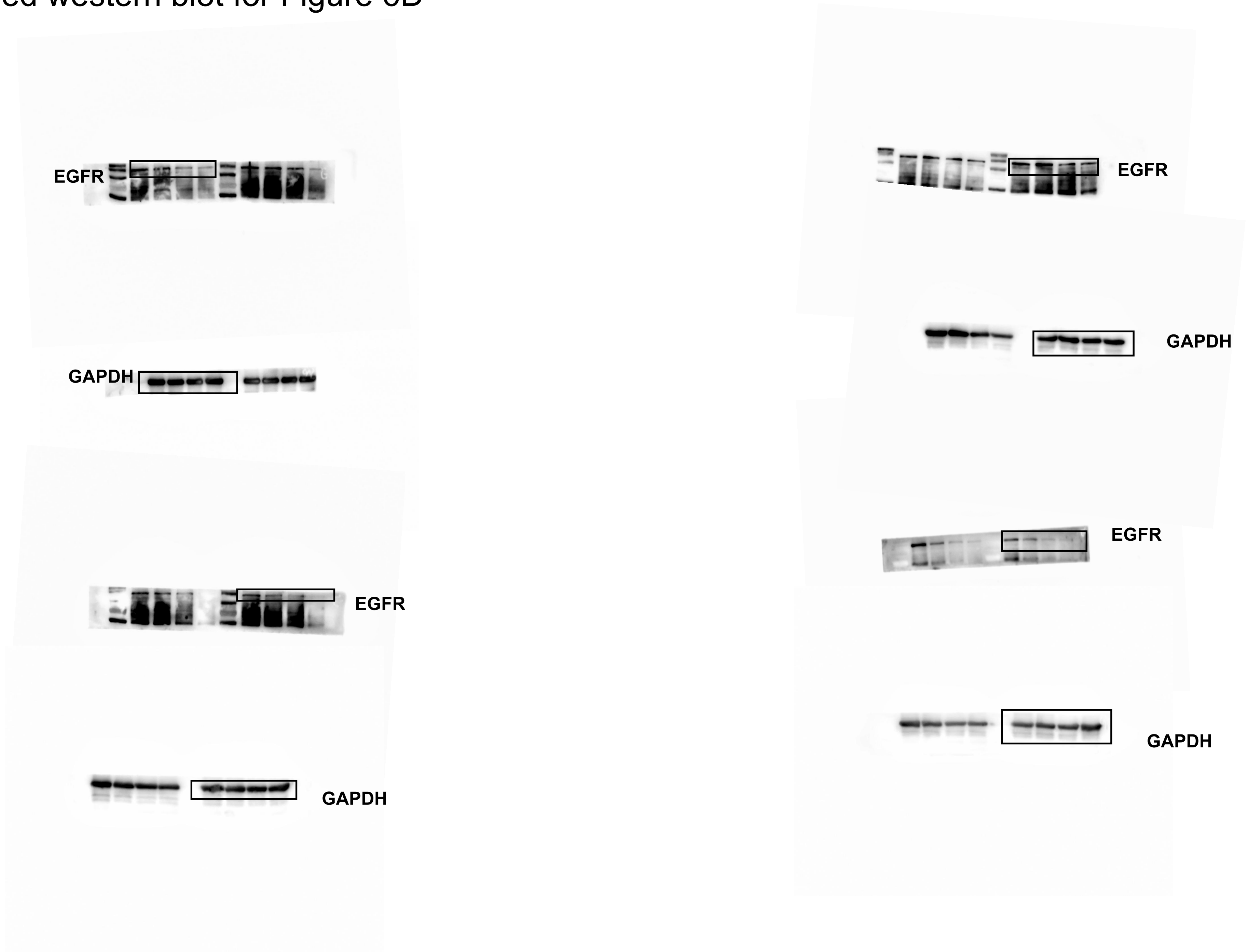

Full and uncropped western blot for Figure 6E

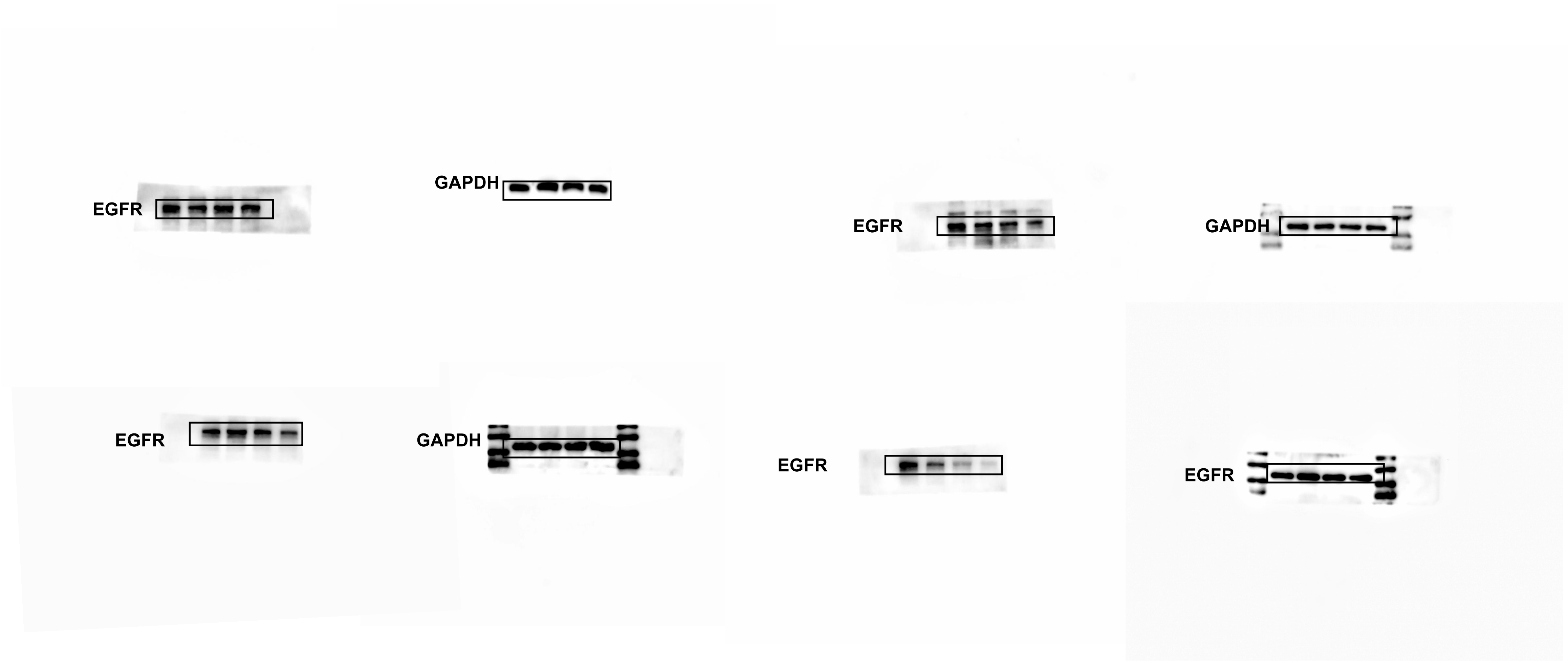

Full and uncropped western blot for Figure 7E

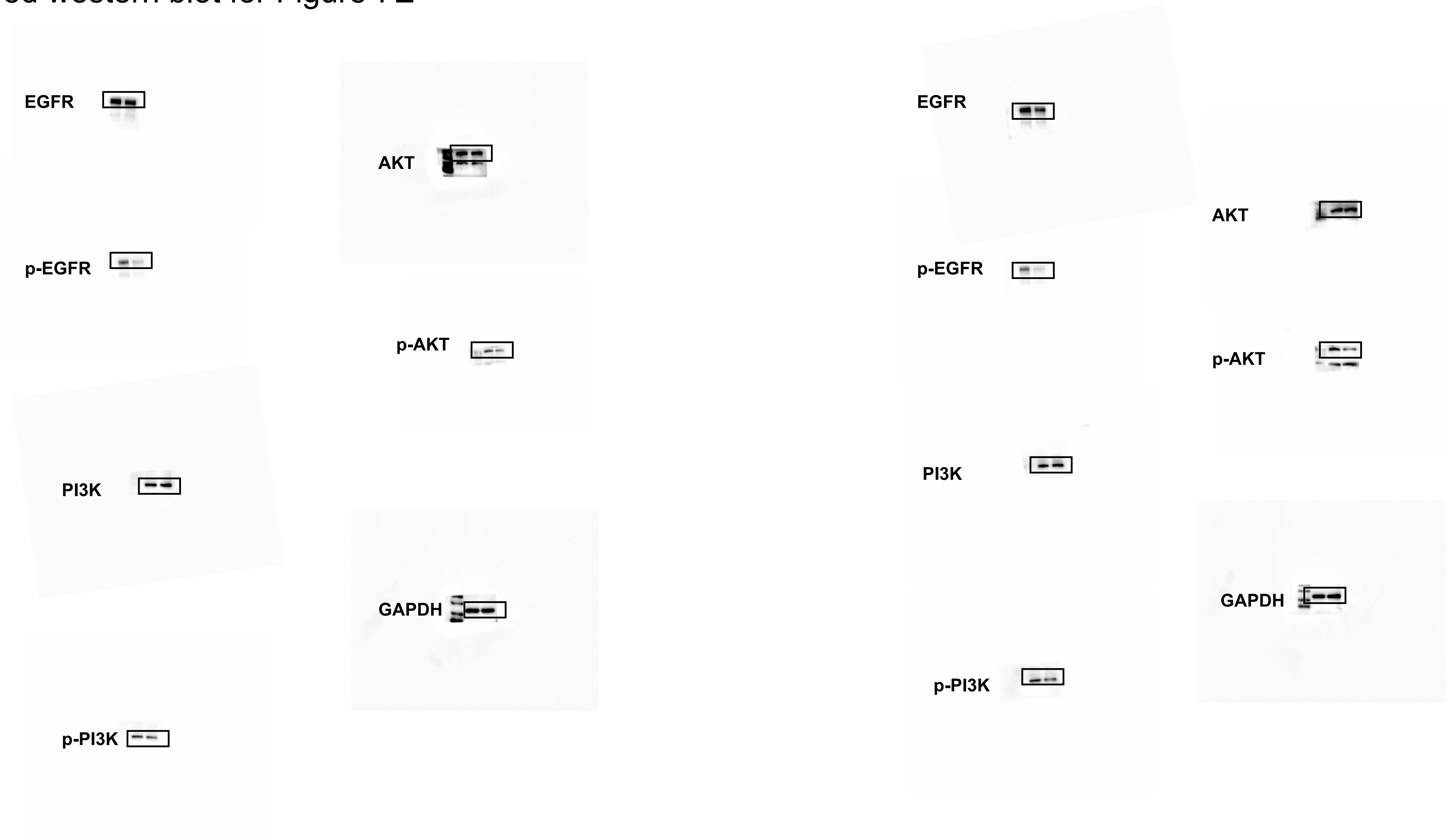

Full and uncropped western blot for Figure S2E

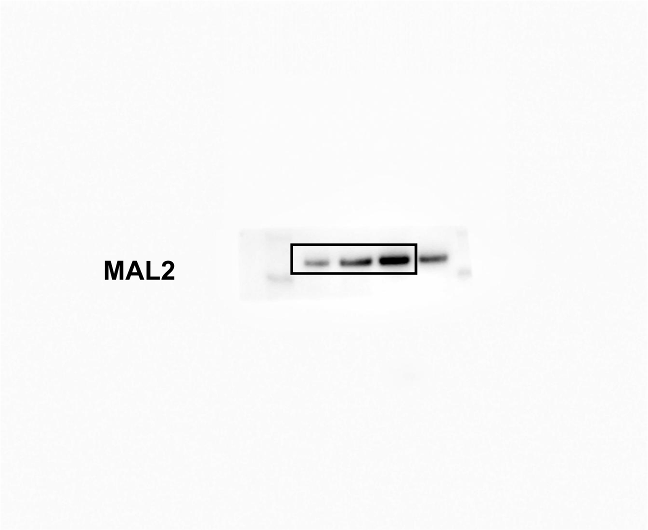

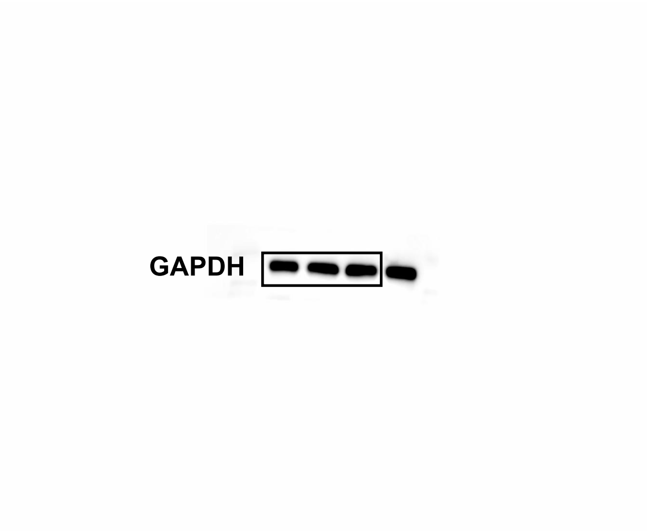

Supplement: Supplementary file 3 — Original data file [file 41419_2024_6775_MOESM3_ESM.pdf]
